# Free‐Breathing Fat Quantification Using a Phase Error‐Corrected Cartesian Acquisition With Spiral Profile Ordering

**DOI:** 10.1002/mrm.70474

**Published:** 2026-06-16

**Authors:** Philipp Braun, Jonathan Stelter, Stefan Ruschke, Johannes M. Peeters, Holger Eggers, Kilian Weiss, Daniela Junker, Dimitrios C. Karampinos

**Affiliations:** ^1^ Institute for Diagnostic and Interventional Radiology, School of Medicine and Health TUM University Hospital, Technical University of Munich (TUM) Munich Germany; ^2^ Philips Healthcare Best the Netherlands; ^3^ Philips GmbH Innovative Technologies Hamburg Germany; ^4^ Philips GmbH Market DACH Hamburg Germany; ^5^ Laboratory of Magnetic Resonance Imaging Systems and Methods Ecole Polytechnique Federale de Lausanne (EPFL) Lausanne Switzerland; ^6^ CIBM Center for Biomedical Imaging (CIBM) Lausanne Switzerland

**Keywords:** Cartesian spiral profile ordering (CASPR), free‐breathing, isotropic resolution, phase errors, proton density fat fraction (PDFF), time‐interleaved multi‐echo gradient echo

## Abstract

**Purpose:**

To develop a phase‐corrected time‐interleaved multi‐echo gradient echo Cartesian acquisition with spiral profile ordering (TIMGRECASPR) for abdominal large‐FOV proton density fat fraction (PDFF) mapping at 3 *T*, demonstrating its sampling flexibility and inherent self‐gating capabilities at high isotropic resolutions.

**Methods:**

A methodological framework was developed to correct eddy current‐ and concomitant gradient‐related phase errors in a TIMGRECASPR acquisition. Sequence parameters were optimized with an XCAT simulation. Validation was conducted in phantoms and in vivo thigh and abdominal imaging. In five healthy volunteers, liver PDFF and T2*, alongside pancreatic PDFF, were compared to a clinical Cartesian breath‐hold vendor technique. Liver PDFF homogeneity was evaluated visually and across five different axial subvolumes. Mean absolute PDFF bias and within‐subject standard deviation (wSD) across repeated scans were quantified. Pancreatic delineation was quantified by line profiles across the tissue boundary.

**Results:**

Short shot lengths were required for a sufficient temporal resolution of the respiratory motion self‐navigation. Without phase corrections, data showed severe peripheral PDFF bias, especially at higher spatial resolutions. In a water phantom, PDFF bias ranged from −5% to 5% over 400 mm in readout direction, successfully mitigated by the proposed phase corrections. Liver PDFF maps exhibited improved homogeneity and accurate quantification results, verified by the breath‐hold reference scan (0.6% PDFF bias, wSD = 0.11%). Pancreatic boundaries were clearly visualized and provided accurate pancreatic PDFF maps (1.1% PDFF bias, wSD = 0.37%).

**Conclusion:**

This work demonstrates the feasibility of a free‐breathing monopolar time‐interleaved gradient echo sequence based on a Cartesian spiral acquisition at 3 *T* when accounting for phase errors.

## 1 Introduction

Proton density fat fraction (PDFF) mapping has proven to be an invaluable tool for assessing abdominal and musculoskeletal diseases [1, 2, 3, 4]. Hepatic and pancreatic steatosis is characterized by an abnormal accumulation of fat inside these organs and is closely linked to metabolic dysfunction [5, 6, 7, 8, 9, 10]. Many recent studies have attempted to characterize both the liver and the pancreas and highlighted the synergy in measuring the PDFF in both organs to provide a more comprehensive metabolic profile [11, 12, 13]. Liver Fat deposition is relatively homogeneous and does not require high resolution imaging [14, 15]. However, the fat deposition in the pancreas is spatially heterogeneous and given the small irregular shape of the organ, high‐resolution imaging is required for pancreas PDFF mapping [14, 16, 17].

Traditionally, chemical shift encoding‐based water‐fat separation methods rely on breath‐hold (BH) acquisitions in the abdomen [5, 18, 19, 20, 21]. However, for many patients a BH scan might not be feasible due to their incapability to remain still or to hold their breath for an extended period [22]. In such cases, free‐breathing acquisitions are preferred, but these require either prospective or retrospective motion correction to mitigate motion‐related artifacts. conventional strategies involve the use of external devices like respiratory belts and optical cameras, the use of navigator echoes for self‐navigation or the use of 2D sequential acquisitions [23, 24, 25, 26, 27].

The use of non‐Cartesian trajectories offers increased robustness to motion, albeit at the cost of greater sensitivity to gradient imperfections. Despite this sensitivity, radial stack‐of‐stars (SoS) acquisition techniques have been employed successfully to obtain accurate PDFF measurements after the correction of these gradient imperfections [22, 25, 28, 29, 30, 31, 32, 33]. This trajectory inherently supports motion self‐navigation due to the oversampling of the k‐space center in every acquisition shot, eliminating the need for any additional navigator echoes [34]. This allows for retrospective motion estimation using a principal component analysis (PCA) without the need for any sequence modifications or additional hardware. However, the radial SoS trajectory may constrain the shot length to the chosen through‐plane resolution and coverage. Acquisitions with high isotropic resolution (below 2.5 mm isotropic resolution) and large through‐plane coverage result in long shot durations, thereby degrading the performance of the motion self‐navigator, which assigns the whole shot to its respective motion state. Consequently, slice thickness of radial SoS acquisitions is typically limited to approximately 5 mm for liver PDFF mapping, while allowing for sufficient spatial coverage [22, 25, 35].

A Cartesian acquisition with spiral profile ordering (CASPR) was shown to be well‐suited to capture rapid dynamics with high spatial resolution and coverage [36, 37]. Its application in body MRI has been mostly limited to qualitative imaging and relaxation mapping [38, 39, 40, 41, 42]. Similar to the radial SoS, the CASPR trajectory can be self‐navigated [36]. In addition, the shot‐length of the CASPR trajectory for sampling the joint two‐dimensional phase‐encoding space remains independent of the chosen spatial resolution and coverage in the readout direction, which allows the use of higher isotropic resolutions while maintaining motion navigation capabilities. This would provide advantages for the visualization of smaller organs, such as the pancreas, which might be challenging to visualize for other sequences due to its size, its proximity to the intestines and its location beneath the diaphragm [16, 17]. Moreover, by setting the readout direction to feet‐head, a large longitudinal FOV can be achieved allowing coverage of multiple anatomies in one scan.

The severity and impact of phase errors are highly dependent on the employed k‐space trajectory. Fat quantification techniques are typically based on either a magnitude‐ [43] or a complex‐based formulation [44]. Complex‐based formulations have the benefit of having a higher noise performance as well as robustness to fat modeling errors [45, 46]. However, this comes at the cost of a greater susceptibility to phase errors. The correction of these errors is necessary to avoid severe quantification artifacts of PDFF mapping especially for lower fat fractions. Common strategies include the use of calibration scans [22, 47, 48], direct measurements via nuclear magnetic resonance probes [49, 50] or by explicitly modeling the expected phase errors [51]. The use of monopolar readouts in a Cartesian acquisition is known to minimize phase error effects. However, appropriate phase error correction strategies need to be adopted especially at 3 *T* where a time‐interleaved acquisition is required to achieve optimal echo time steps for water‐fat separation while using monopolar readouts [52].

In this work, we demonstrate the feasibility of a phase error‐corrected self‐navigated time‐interleaved multi‐echo gradient echo Cartesian acquisition with spiral profile ordering (TIMGRECASPR) for PDFF quantification in simulations, phantoms, and in vivo at high isotropic resolutions at 3 *T*. Phase corrections accounting for eddy current and concomitant gradient‐related phase errors are incorporated in this study. An optimal echo time step for high isotropic resolution imaging at 3 *T* is achieved by employing a monopolar time‐interleaved multi‐echo gradient echo (TIMGRE) acquisition [52].

## 2 Methods

### 2.1 Acquisition Scheme

**FIGURE 1:**
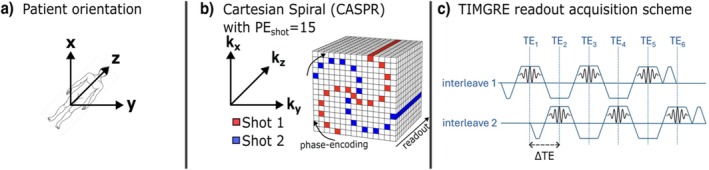
Cartesian sampling with spiral profile ordering performed with a TIMGRE acquisition scheme. The scanner coordinate system is used here. (a) Subject orientation. (b) Visualization of the CASPR trajectory with PE_shot_ = 15 for two consecutive spiral in/out shots separated by the golden angle. The readout direction is given by the *k*_*z*_‐axis whereas phase‐encoding is performed in the $k_{x}-k_{y}$ plane. The k‐space center is sampled in each shot leading to the self‐navigation capabilities of the trajectory. (c) TIMGRE acquisition scheme. Each readout is acquired in two separate interleaved TRs hence doubling the effective TR. In each of these two interleaves, three echoes are acquired using monopolar gradients. Hence, six echoes are obtained in total.

A TIMGRECASPR acquisition scheme is used in this work. The corresponding pulse sequence is shown in Figure 1 [36, 37]. In the following, the scanner coordinate system will be used where the origin is defined to be at the isocenter of the scanner and is right‐handed. The *z*‐axis is parallel to the scanner bore while the *x*‐ and *y*‐axis span the plane perpendicular to the bore (see Figure 1). The readout of the CASPR trajectory is performed along the *k*_*z*_‐axis while phase‐encoding occurs in the $k_{x}-k_{y}$ plane. Readout directions are selected such that the center *k*_*z*_ line is sampled in each shot. This continuous sampling enables motion self‐navigation along the projection in *z*‐direction where we expect the bulk of the motion displacement. The trajectory is acquired in a TIMGRE which acquires echoes in multiple TRs. This enables an optimal echo time‐step for accurate complex‐based water‐fat separation at high spatial resolutions at 3 *T* [52].

### 2.2 Theory of Phase‐Corrected Water‐Fat Separation

Assuming a common $R_{2}^{*}$ value for water and fat, the obtained complex‐based water‐fat signal is given by [43, 45, 53].

(1) $$S\left(t_{n}\right)=\left(W+F\sum_{p=1}^{P}\alpha_{p}e^{i2\pi f_{p}t_{n}}\right)e^{i2\pi f_{B}t_{n}}e^{-R_{2}^{*}t_{n}}=\rho e^{\mathrm{i\varphi}}$$

where *W* and *F* denote the complex water and fat signal, respectively, $\alpha_{p}$ and $f_{p}$ represent the relative amplitude and frequency shift of the *p*‐th fat peak of the employed fat model compared to the water peak, $t_{n}$ is the measured echo time, $f_{B}$is the $B_{0}$‐induced frequency offset, $R_{2}^{*}$ is the real‐valued observed relaxation rate, ρ is the magnitude and φ is the phase of the signal. In the presence of phase errors $\Phi_{\text{total}}$ Equation (1) becomes

(2) $$S\left(t_{n}\right)=\rho e^{i\left(\varphi +\Phi_{\text{total}}\right)}$$

In this work the total phase error is approximated by a linear phase in readout direction (frequency encoding direction) and a concomitant phase resulting from the gradient echo trains [52].

(3) $$\Phi_{\text{total}}=\Phi_{\mathrm{lin}}\left(z,t_{n}\right)+\Phi_{\text{concom}}\left(x,y,z,t_{n}\right)$$

where $\Phi_{\mathrm{lin}}\left(z,t_{n}\right)$ denotes the linear phase of the *n*‐th echo and $\Phi_{\text{concom}}\left(x,y,z,t_{n}\right)$ denotes the concomitant phase accumulated at location (*x, y, z*) for the *n*‐th echo.

#### 2.2.1 Linear Phase $\Phi_{\mathrm{lin}}$

Eddy currents and gradient delays introduce primarily a linear phase in image space along the frequency encoding direction of the monopolar acquisition. According to the Fourier shift theorem, these shifts can be represented as a constant offset in k‐space. These misalignments can be corrected for by acquiring opposite readout polarities per echo [54, 55, 56]. Consequently, a total of 12 calibration central k‐space lines were acquired in this work. These k‐space lines comprise six opposite‐polarity pairs corresponding to each of the six acquired interleaved echoes. The *k*‐space offset between corresponding pairs of lines is used to correct the observed linear phase discrepancy. This offset is determined via the *k*‐space cross‐correlation of the respective calibration line pairs, which are zero‐padded prior to correlation. The linear phase is first estimated for each coil independently; the mean of these estimates is then applied to all coil channels as a common linear phase.

#### 2.2.2 Concomitant Phase $\Phi_{\text{concom}}$

Gradient echo trains generate concomitant gradient fields $\Delta \Phi_{C}\left(x,y,z,t\right)$ which can be described as [52, 57]:

$$\Delta \Phi_{C}\left(x,y,z,t\right)=A\left(t\right)z^{2}+B\left(t\right)\left(x^{2}+y^{2}\right)+C\left(t\right)\mathrm{xz}+D\left(t\right)\mathrm{yz}$$

$$A\left(t\right)=\frac{\gamma }{2B_{0}}\int_{0}^{t}\;\left(G_{x}{\left(t^{\prime }\right)}^{2}+G_{y}{\left(t^{\prime }\right)}^{2}\right)dt^{\prime }$$

$$B\left(t\right)=\frac{\gamma }{8B_{0}}\int_{0}^{t}\;G_{z}{\left(t^{\prime }\right)}^{2}dt^{\prime }$$

$$C\left(t\right)=-\frac{\gamma }{2B_{0}}\int_{0}^{t}\;G_{x}\left(t^{\prime }\right)G_{z}\left(t^{\prime }\right)dt^{\prime }$$

(4) $$D\left(t\right)=-\frac{\gamma }{2B_{0}}\int_{0}^{t}\;G_{y}\left(t^{\prime }\right)G_{z}\left(t^{\prime }\right){\mathrm{dt}}^{\prime }$$

where $G_{x}$, $G_{y}$, and $G_{z}$ denote the applied gradient waveforms. Concomitant gradient induced phase errors get stronger with increasing distance from the isocenter as well as with increasing echo time. In single TR readouts, the accumulated phase during the echo train would be linear. This leads to a bias in the estimation of the main field inhomogeneity usually irrelevant for water‐fat separation. However, for time‐interleaved sequences the phase evolution is modulated by the employed interleave scheme which would introduce a PDFF quantification bias due to concomitant gradient effects [52]. Hence, these errors need to be corrected in time‐interleaved acquisitions, where the phase correction is computed from the known applied gradient waveforms. In contrast to the linear phase correction, the correction for concomitant gradient induced phase errors does not rely on any measured data. Deviations from the applied gradient waveforms due to eddy currents are assumed to be negligible at 3 *T* and are not considered within the scope of this work.

### 2.3 Image Reconstruction and Water‐Fat Separation

**FIGURE 2:**
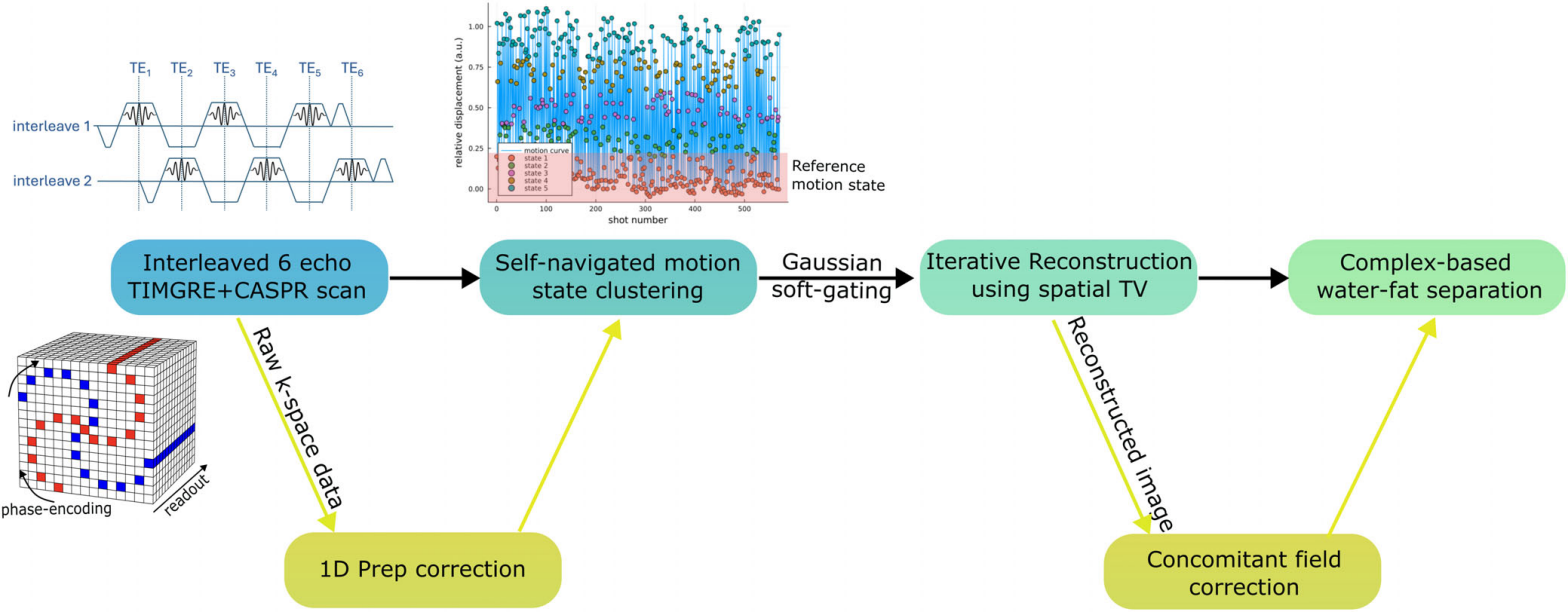
Proposed reconstruction pipeline for a time‐interleaved six‐echo CASPR acquisition with self‐navigated motion compensation. The 1D Prep correction is correcting for eddy current‐induced phase errors along the readout direction. Raw data is motion self‐navigated into 5 motion bins using only their magnitude profiles to avoid errors due to *B*_*0*_ signal drift. The iterative reconstruction uses soft gating and a spatial TV term. Soft‐gating weights are derived using a Gaussian weighting function where the variance was chosen to correspond to the distance between the reference state (end‐expiration) and the second closest state. The reconstructed images are corrected for concomitant gradient‐induced phase errors. Complex‐based water‐fat separation is performed using a multi‐resolution graph‐cut algorithm employing a multi‐peak fat model and a common *T*_2_* decay.

The reconstruction pipeline for generating PDFF maps from a monopolar time‐interleaved multi‐echo CASPR acquisition is illustrated in Figure 2.

The acquired one‐dimensional calibration data is used to calculate the linear phase term Φ_lin_, which is directly applied in image space of the Fourier transformed k‐space data. The corrected data is self‐navigated into five different motion states using a PCA of the projection magnitude 1D profiles. Soft‐gating weights are derived using a Gaussian weighting function where the variance was chosen to correspond to the distance between the reference state (end‐expiration) and the second closest state. All data is used for the reconstruction. The iterative reconstruction solves the following inverse problem for the chosen soft‐navigated motion state:

(5) $$\;\hat{m}={\text{argmin}}_{m}{\left\|\mathrm{WF}\mathrm{Sm}-d\right\|}_{2}^{2}+\lambda \;{\mathrm{TV}}_{\text{spatial}}\left(m\right)$$

where W is a weighting parameter containing the sampling density compensation multiplied with the calculated soft‐gating weights, F is the Fourier transform, *S* are the coil sensitivity maps derived from a pre‐scan, *d* is the sampled k‐space and *m* and $\hat{m}$ are the unknown and estimated image respectively. A spatial total variation term ${\mathrm{TV}}_{\text{spatial}}$ regularization term is included. The ADMM solver is used for the minimization problem in Equation (5). The reconstructed data $\hat{m}$ is corrected for phase errors induced by concomitant gradients, using the analytical formulas given in Equation (4). Water‐fat separation is performed using a multi‐resolution graph‐cut algorithm assuming a common *R*_2_* value [43, 44, 58]. The Berglund 10‐peak fat model is used to model the spectral complexity of the fat peaks for 3 *T* [59]. For phantoms, the self‐navigation part of the reconstruction is omitted and the soft‐gating weights are instead set to one.

### 2.4 Measurements

A 3 *T* Ingenia Elition X system (Philips Healthcare, Best, The Netherlands) was used for all scans. All scans were performed with a monopolar interleaved six‐echo CASPR sequence with two interleaves containing three echoes each, sagittal slice orientation, flip angle of 3°, pseudo golden angle ordering [60], and 45° spiral twist degree using a spiral‐in/out trajectory. The sequence parameters for phantom and in vivo measurements are shown in Table 1.

**TABLE 1:** Imaging parameters used in the phantom and in vivo measurements.

	Phantom		In vivo		
	Water phantom	Calimetrix	Thigh	Abdomen	
Trajectory	TIMGRECASPR	TIMGRECASPR	TIMGRECASPR	Cartesian	TIMGRECASPR
Shot length (ms)	437	—	351 438	—	351
Scan mode	3D	3D	3D	3D	3D
FOV [*x*, *y*, *z*] (mm^3^)	210 × 400 × 400	200 × 200 × 200	250 × 400 × 250	300 × 400 × 150	250 × 400 × 250
Voxel size [*x*, *y*, *z*] (mm^3^)	Isotropic 1.5	Isotropic 1.5	Isotropic 1.5 Isotropic 2.5	2 × 3 × 6	Isotropic 2.5
Echoes	6	6	6	6	6
TE1/dTE (ms)	1.19/1.0	1.25/1.0	1.19/1.0 0.98/0.8	1.35/1.1	0.98/0.8
TR (ms)	7.7	7.9	7.8 6.2	7.8	6.2
FA *α* (°)	3	3	3	3	3
Respiratory compensation	—	—	—	Breath‐hold	Self‐navigated soft‐gating
Acceleration	—	—	—	CS‐SENSE *R* = 4	—
Total scan time	9 min 12 s	4 min 28 s	10 min 56 s 3 min 12 s	9.4 s	3 min 12 s

*Note:* The thigh scan was performed twice for two different isotropic voxel sizes.

#### 2.4.1 XCAT Simulation

Simulations using the extended cardiac‐torso (XCAT) phantom modeled physical properties such as PDFF, T2* relaxation and magnetic susceptibility at 3 *T* [61]. The pancreatic PDFF was set to 10% and a sinusoidal respiratory motion was included (respiratory_period = 5 s, max_diaphragm_motion = 2 cm, max_AP_extension = 1 cm). Different numbers of phase encodes per shot (PE_shot_) 25, 50, and 100 leading to shot lengths of 311, 622, and 1244 ms were simulated (2.5 × 2.5 × 2.5 mm^3^, TR/TE_1_/∆TE = 12.44/0.98/0.8 ms). PE_shot_ refers to the number of phase‐encoding steps (k‐space lines) acquired in each spiral in/out trajectory (see Figure S1 for representative phase‐encoding trajectories and corresponding point spread functions [PSF]). Total scan time, spatial FOV and resolution were kept constant across all three simulated PE_shot_. Consequently, simulations using higher PE_shot_ acquired fewer phase‐encoding spirals in total, with the PE_shot_ and total number of acquired spiral being inversely proportional. Both interleaves are acquired in immediate succession for each (*k*_*x*_, *k*_*y*_) encoding line.

#### 2.4.2 Simulation

A large FOV corresponding to the later used liver FOV (FOV 250 × 400 × 250 mm^3^) was simulated, where every voxel was modeled by a water‐only signal model. The signal model corresponds to Equation (1) with the fat component set to zero. The concomitant gradient field terms $\Delta \Phi_{C}\left(x,y,z,t\right)$ from Equation (4) were calculated for a set of different echo time combinations: ∆TE = 0.8/0.9/1.0 ms and corresponding TE_1_ = 1.0/1.1/1.2 ms. These concomitant gradient errors were then modeled as an additional phase contribution in the water‐only signal equation. Water‐fat separation was then performed using the proposed multi‐resolution graph‐cut algorithm.

#### 2.4.3 Phantom Measurements

A large water phantom (FOV 210 × 400 × 400 mm^3^) as well as a custom water‐fat phantom (Calimetrix LLC, Wisconsin‐Madison, USA) (FOV 200 × 200 × 200 mm^3^) containing fat vials with fat fractions of 0%, 3.5%, 6.2%, 9.0%, 12.1%, 17.3%, 23.0%, 33.4%, 43.2%, 53.3%, 63.4%, 73.1%, 82.5%, 90.8%, and 100% PDFF were used to evaluate and characterize occurring phase errors and assess the effectiveness of the proposed corrections. Different fat concentrations were achieved by a varying mixture of distilled water and peanut oil. The PDFF values of the water‐fat phantom were validated using a fully sampled Cartesian TIMGRE scan with 1.4 mm isotropic resolution, which was also verified by single‐voxel magnetic resonance spectroscopy measurements. For both phantoms, data was acquired at an isotropic voxel size of 1.5 mm and ∆TE = 1.0 ms.

#### 2.4.4 In Vivo Measurements

Five different healthy volunteers were scanned for this work (mean age 29.0 ± 3.4 years, three female, mean weight 62.2 ± 5.3 kg). A thigh scan from one volunteer and free‐breathing abdominal scans from five different volunteers were obtained. The study was approved by the local institutional review board (Klinikum rechts der Isar, Technical University of Munich, Munich, Germany) and informed consent was given by all volunteers. The thigh scan was used to evaluate the performance of the phase correction steps in vivo without the influence of any motion artifacts. In both scans, calibration lines for the linear phase correction were acquired in a short BH to minimize motion‐related interference with the correction procedure. Calibration lines were acquired in readout direction for each interleaved echo. The readout direction was set to feet‐head to enable self‐navigation using the CASPR trajectory and to allow for a wide coverage of the abdominal region in the superior–inferior direction. ∆TE = 1.0 ms and ∆TE = 0.8 ms were evaluated for the thigh (FOV 250 × 400 × 250 mm^3^, isotropic voxel size 1.5 mm/2.5 mm), given the dependence of the phase errors on the employed echo times. Liver scans were performed with ∆TE = 0.8 ms to reduce shot length and its influence on the motion self‐navigator (FOV 250 × 400 × 250 mm^3^, isotropic voxel size 2.5 mm, TR/TE_1_/∆TE = 6.2/0.98/0.8 ms, scan time = 3 min 13 s, PE_shot_ = 25, shot length = 351 ms). The exact shot lengths applied during the acquisition were extracted directly from the scanner configuration files. All abdominal scans were performed thrice to assess short‐term repeatability via the within‐subject standard deviation (wSD).

An additional abdominal reference BH Cartesian scan was performed. This scan uses a similar (*x*, *y*) resolution compared to the TIMGRECASPR sequence (FOV 300 × 400 × 150 mm^3^, voxel size 2 × 3 × 6 mm^3^, scan time BH = 10 s) and performs PDFF quantification online based on the vendor's product implementation (mDixon Quant). For each volunteer, the mean bias was calculated between the BH reference and the repeated TIMGRECASPR acquisitions. From these individual biases, the overall mean absolute bias is determined.

Whole‐liver segmentation was performed using the VIBESegmentator, an open‐source deep learning model for full‐torso segmentation with reported DICE coefficients for liver segmentations being between 0.93 and 0.94 when compared to other automatic or manual segmentations [62]. To systematically evaluate the PDFF in different regions of the liver, the segmentation was partitioned into five axial cross‐volumes of equal thickness along the feet‐head direction (Figure S2). These were analyzed separately in the Supporting Information.

Pancreatic segmentations were also performed using the VIBESegmentator with DICE coefficients being reported to be between 0.73–0.81 when compared to other automatic or manual segmentations. Pancreatic sharpness was evaluated using the slope along a 1D line profile across the pancreatic boundary. Pancreatic PDFF measurements were analyzed separately in the Supporting Information.

## 3 Results

### 3.1 XCAT Simulation Results

PCA‐derived motion curves from XCAT simulations for the three tested PE_shot_ demonstrated that only PE_shot_ = 25 (corresponding to a shot length of 311 ms) successfully resolved the respiratory cycle, whereas a higher PE_shot_ failed to adequately capture the whole extent of the respiratory sinusoidal motion with a breathing period of 5 s. PCA‐derived motion curves are displayed in Figure S3.

**FIGURE 3:**
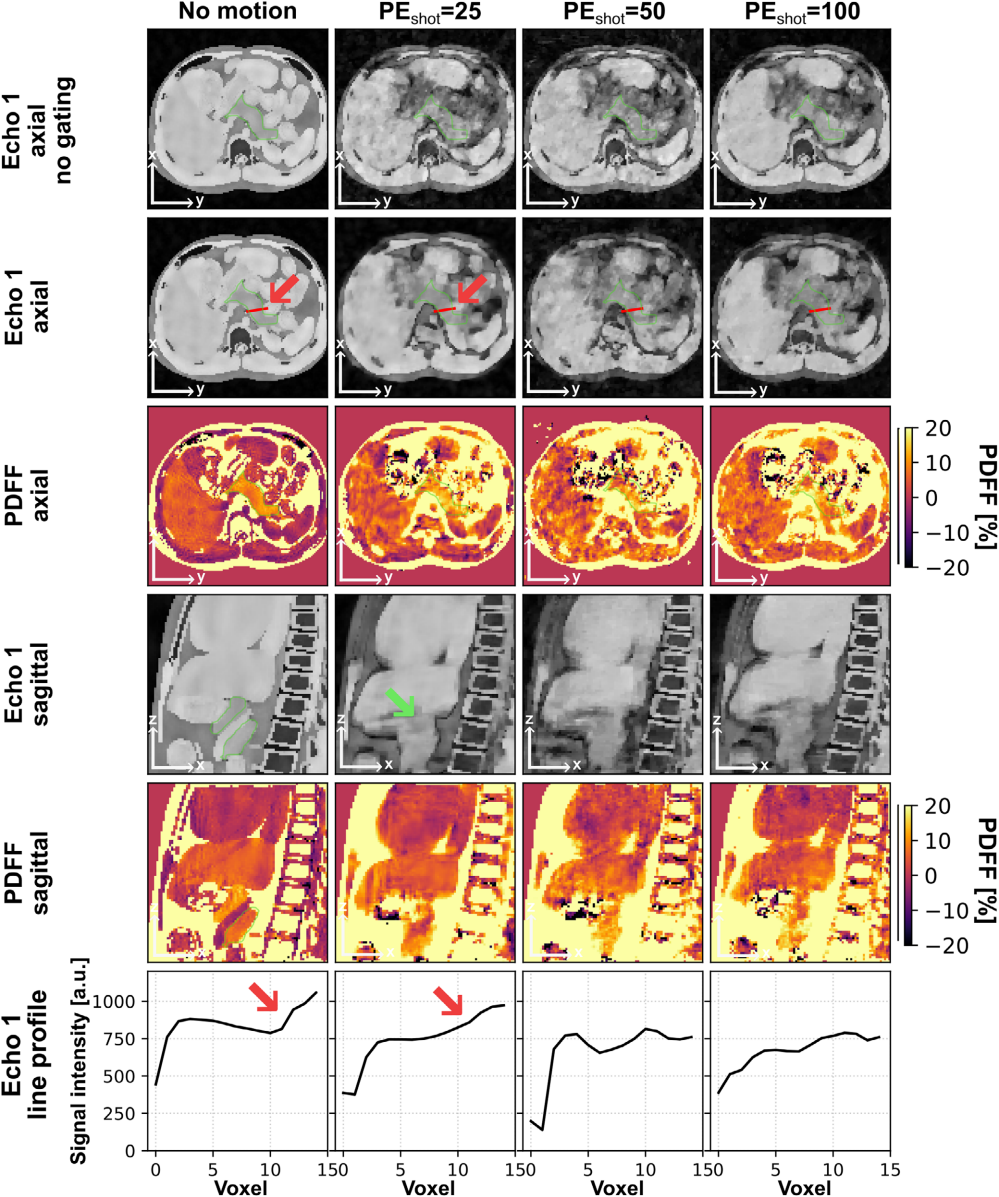
XCAT phantom simulation results when no motion was added and when a respiratory motion with a respiratory cycle of 5 s was added to an acquisition using PE_shot_ 25, 50 and 100. The pancreas has a uniform PDFF of 10%. The first row shows results without motion navigation. The later rows use a self‐navigated reconstruction with motion curves displayed in Figure S3. The green contour highlights the pancreas outline visible in the first echo image of the motion‐unaffected simulation. Mean pancreatic PDFF within axial contour from left to right 9.75% ± 2.01%; 11.3% ± 2.9%; 11.2% ± 5.2%; 11.5% ± 5.0%. In the echo 1 sagittal view of PE_shot_ = 25 the pancreas outline is still visible (green arrow). 1D intensity profiles along the red line drawn in the echo 1 axial image are displayed in the last row. For PE_shot_ = 25 internal blurring is minimized, and edge sharpness is best preserved, enabling a clearer delineation of the pancreatic borders similar to the non‐motion affected simulation. The red arrows highlight the boundary to a surrounding anatomy with higher signal intensity, which is only effectively resolved when PE_shot_ = 25.

Corresponding echo and PDFF images after using a soft‐navigated reconstruction are presented in Figure 3. Axial PDFF maps clearly show a superior pancreas delineation for PE_shot_ = 25, as also confirmed by the accompanying 1D intensity profile across the organ. Mean pancreatic PDFF inside the axial contour shows a smaller standard deviation for PE_shot_ = 25.

### 3.2 Simulation Results

Simulated PDFF errors due to concomitant gradients on a 0% true PDFF are shown in Figure S4. Concomitant errors mainly manifest as concentric rings in the *x–y* plane around the isocenter. Only a small bias is observed in the *z* direction parallel to the scanner bore. For higher resolutions and thus also higher ${\mathrm{TE}}_{1}$/ΔTE combinations a stronger PDFF quantification error can be observed which increases to up to 1.5% for voxels 20 cm away from the isocenter.

### 3.3 Phantom Results

**FIGURE 4:**
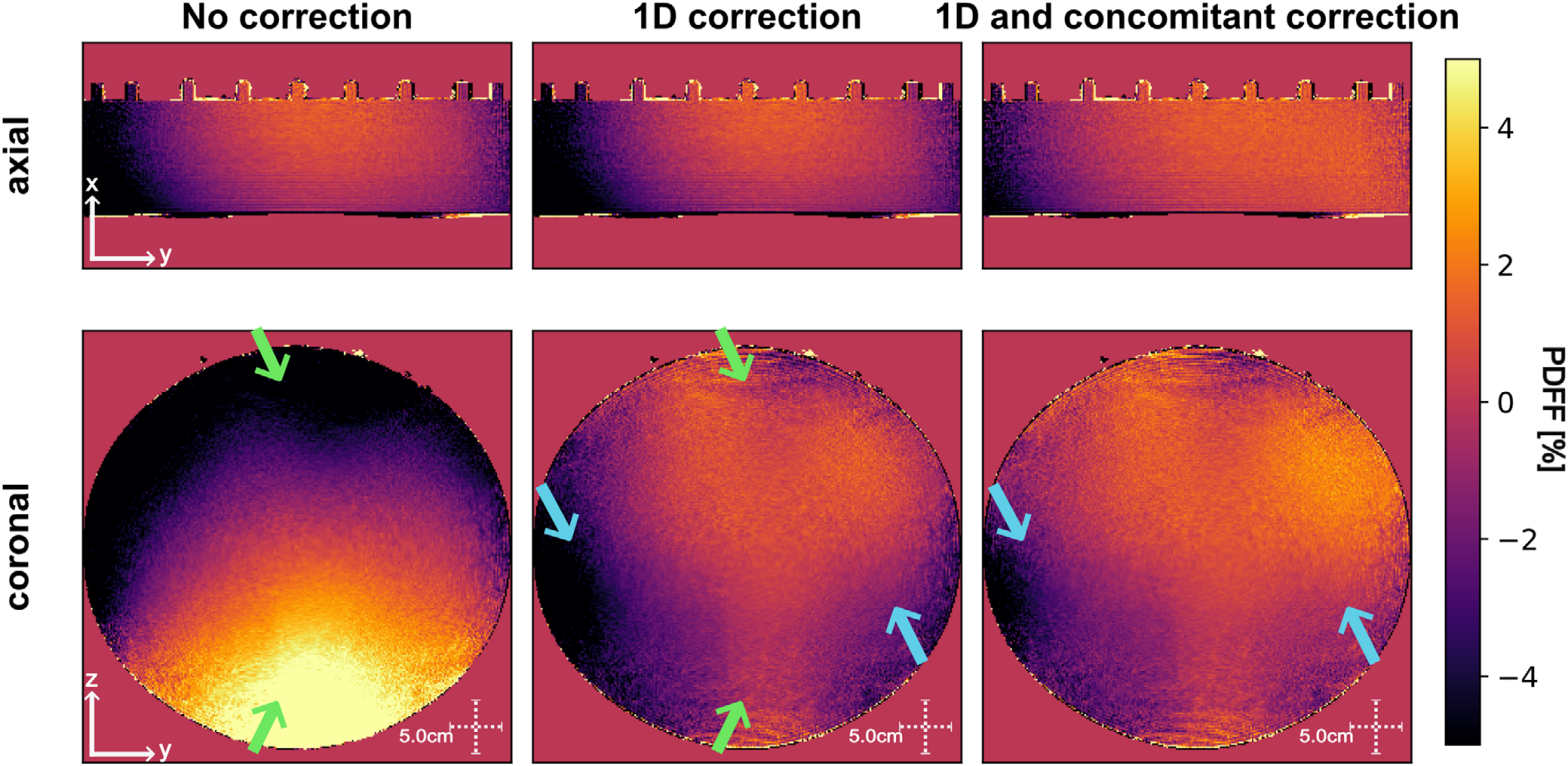
PDFF maps of a large water phantom (FOV 210 × 400 × 400 mm^3^) after no phase corrections, only the linear phase correction, and both the linear as well as a concomitant gradient correction. PDFF maps are shown in central axial and coronal planes. Linear PDFF bias in *k*_*z*_ is reduced after the proposed linear phase correction (PDFF at green arrows before correction: −6.1% to 7.5% PDFF; after correction 0.49% to 0.38% PDFF). The concomitant phase correction can remove most of the remaining errors in the periphery (PDFF at blue arrows before correction: −4.3% and −2.8% PDFF; after correction −1.7% and −0.4% PDFF).

PDFF maps of the large water phantom (Figure 4) show a large linear PDFF bias in the readout direction. Errors range from −5% to 5% PDFF at the periphery of the phantom. After correcting for the linear phase $\Phi_{\mathrm{lin}}$, this PDFF bias is largely removed. However, a small PDFF bias remains at the periphery of the phantom mainly visible in the *y* direction. Adding the concomitant correction reduces these artifacts and provides a more spatially homogeneous PDFF map.

**FIGURE 5:**
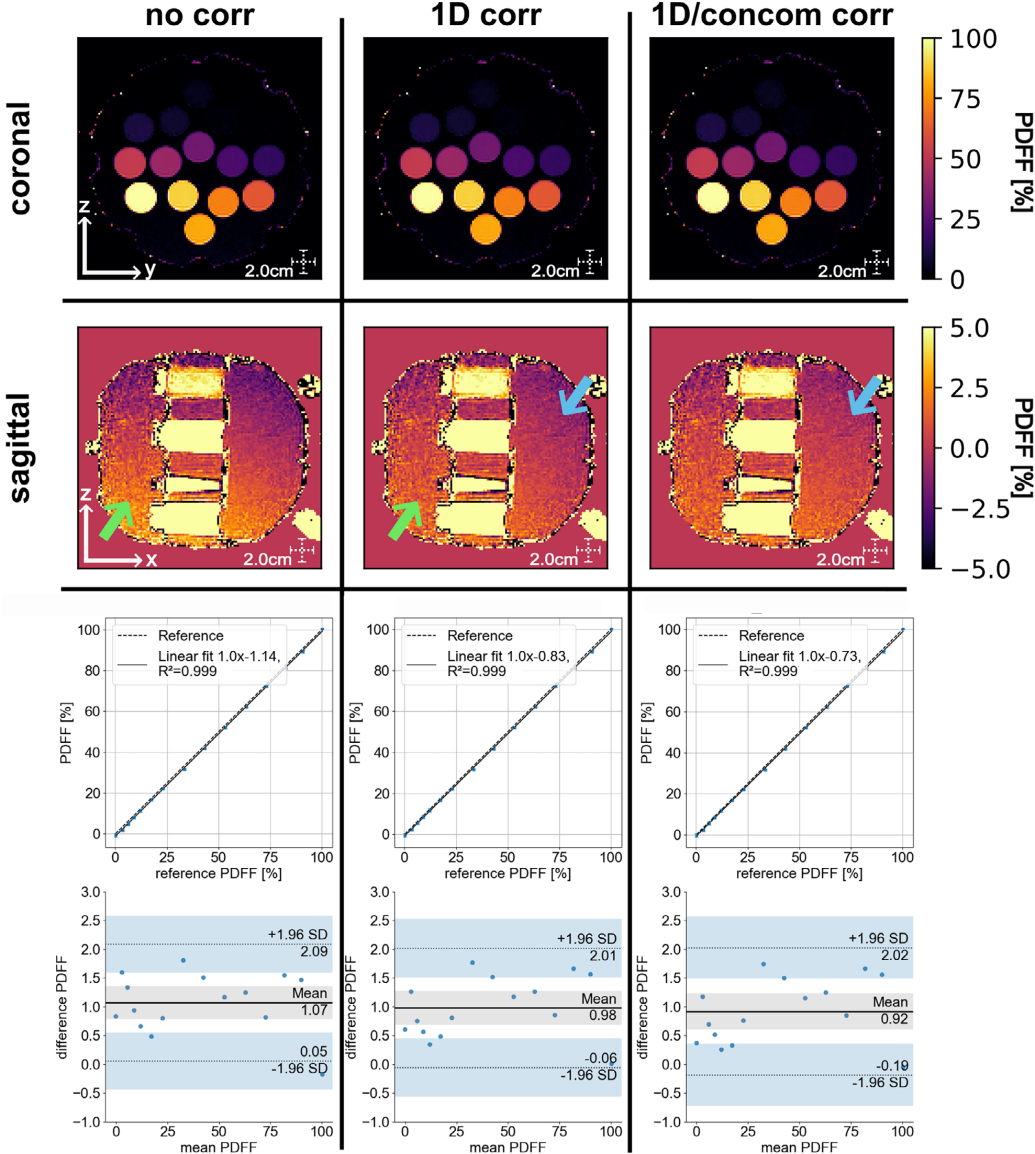
PDFF quantification results for a water‐fat phantom (FOV 200 × 200 × 200 mm^3^) containing 15 fat vials with varying fat fractions ranging from 0% to 100% PDFF. The coronal view shows the arrangement of the fat vials inside the water‐filled phantom. The sagittal view is centered around low PDFF fractions to highlight PDFF errors due to phase errors. Errors due to linear or concomitant gradient‐induced phase are marked with green and blue arrows, respectively. The bottom row shows a comparison to a previous reference scan (Cartesian fully sampled TIMGRE, 1.4 mm isotropic) which was also verified by a MRS scan. Additionally Bland–Altmann plots are shown to highlight the improved quantification results of the phase‐corrected PDFF maps. The one‐dimensional linear phase correction reduces PDFF errors in readout direction, the concomitant gradient‐induced phase correction eliminates some of the remaining local bias in the periphery.

Figure 5 shows quantification results for the water‐fat phantom. The top row visualizes the axial positioning of the fat vials in the water background. The second row shows a sagittal slice through the phantom for a much tighter PDFF window. Without corrections one can again observe a linear PDFF bias in the readout direction *k*_*z*_ which ranges from −2.0% to +3.2% PDFF bias inside the full extent of the phantom. This bias gets reduced to a range between −0.3% to 1.5% after the inclusion of the linear phase correction (shown by green arrows). Some residual errors remain in the periphery (marked by blue arrows) which gets decreased after the implementation of the concomitant phase correction. The Bland–Altmann plots generally show a good agreement in the fat vials to the previously acquired high resolution reference TIMGRE scan. This is particularly the case for the phase‐corrected map, where the mean error inside the fat vials gets reduced to around 0.9% from 1.1% for the uncorrected map.

### 3.4 In Vivo Results

**FIGURE 6:**
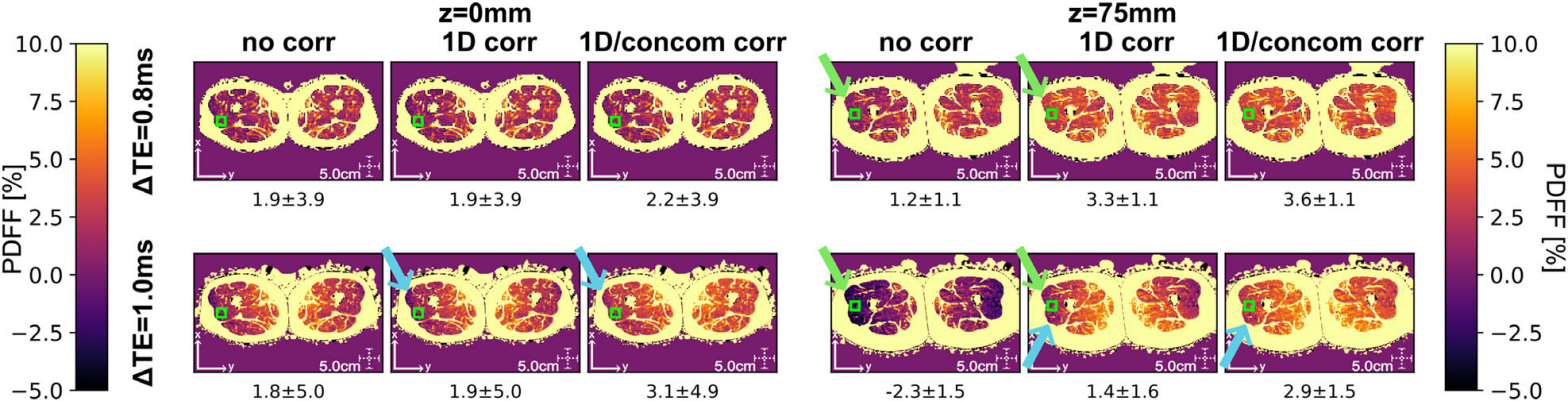
PDFF maps from a volunteer thigh scan (FOV 250 × 400 × 250 mm^3^). First row shows results for *∆TE* = 0.8 ms whereas the second row shows results for *∆TE* = 1.0 ms. The first three columns show results for the central axial slice, the last three columns for a slice toward the periphery of the image. Each slice was evaluated after a complex‐based reconstruction without phase corrections, with only the linear phase correction, and after both the linear as well as a concomitant phase correction. Green arrows show locations which were improved after the linear phase correction. Blue arrows show locations where the phase correction for concomitant phase errors improved the image quality. *∆TE* = 1.0 ms was affected more strongly by phase errors. Linear phase errors are causing strong PDFF bias at the edge of the FOV in *z*.

Thigh scans were performed for two different *∆TE* = 1.0 ms/0.8 ms (Figure 6). For *∆TE* = 0.8 ms PDFF errors due to phase errors are generally small. However, for slices further away from the isocenter a strong linear PDFF bias can be observed which is mitigated successfully by the proposed correction. For *∆TE* = 1.0 ms, errors due to the linear phase and concomitant effects are larger. In the peripheral slices of the thigh, the negative PDFF bias inside the drawn ROI is reduced. In these ROIs PDFF values change from −2.3% ± 1.5% to 2.9% ± 1.5%.

**FIGURE 7:**
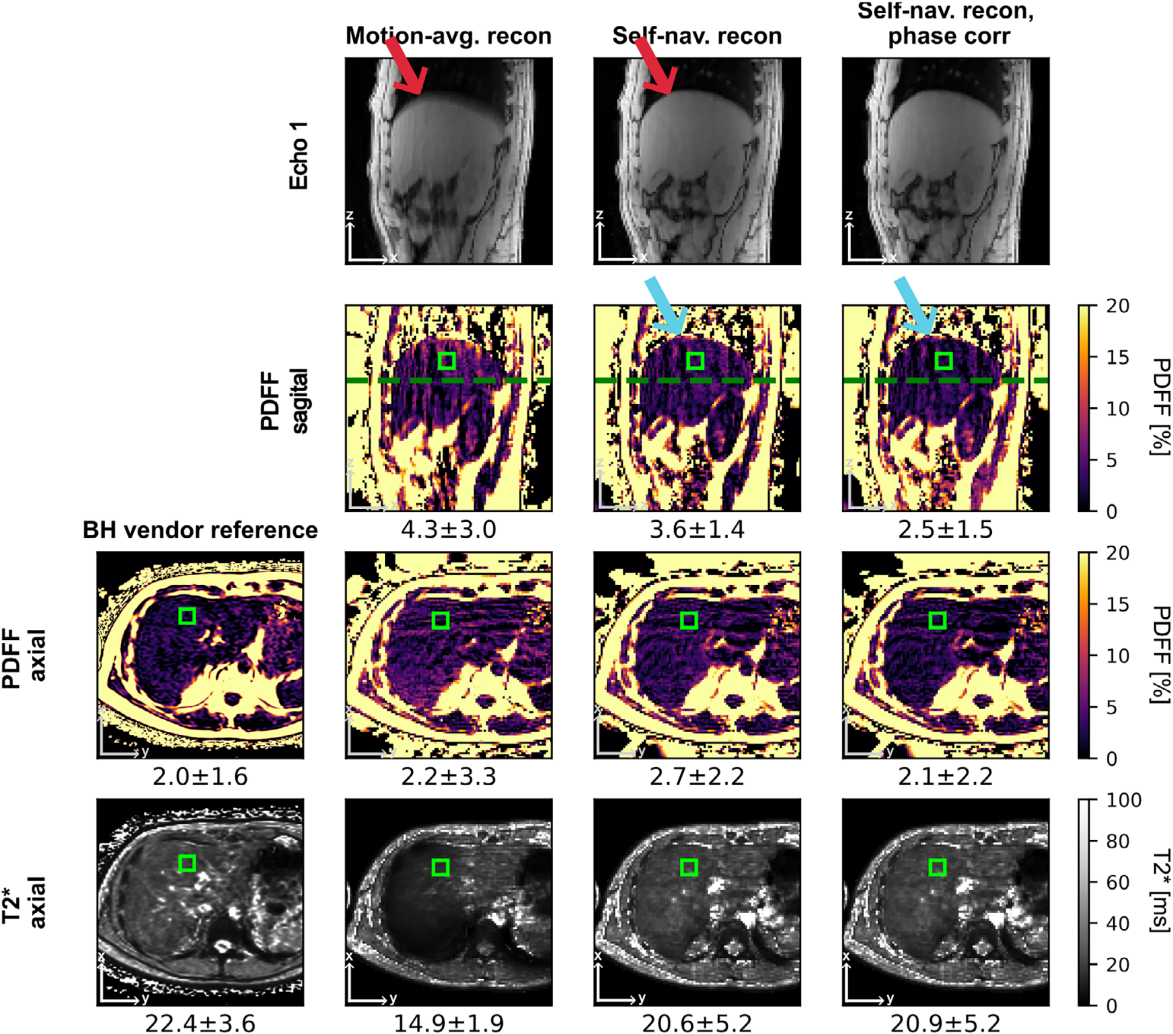
Liver first echo magnitude images, PDFF maps for a sagital slice as well as PDFF and *T*_2_* maps for an axial slice along the dashed line (FOV 250 × 400 × 250 mm^3^). From left to right: BH reference scan, motion‐averaged reconstruction, self‐navigated reconstruction, and self‐navigated reconstruction with the proposed linear and concomitant phase correction steps. The applied self‐navigation removes ghosting artifacts and provides sharp echo images (red arrows). The proposed phase corrections are successful in removing PDFF bias as highlighted by the decrease of PDFF close to the liver dome (blue arrows). PDFF and *T*_2_* quantification at the drawn ROIs of the self‐navigated and phase‐corrected reconstruction are similar to the BH reference scan. Striation patterns are visible in the TIMGRECASPR echo 1 images and PDFF maps.

Five different volunteer abdominal scans were performed with the proposed TIMGRECASPR sequence. Figure 7 shows the results for one volunteer. The effects of the self‐navigated reconstruction as well as the phase corrections are shown and PDFF and *T*_2_* maps are compared to a BH reference scan. The use of the self‐navigated reconstruction with soft gating weights provides sharp echo images and the liver dome is clearly visible (red arrows). *T*_2_* maps show similar values to the BH reference scan (20.9 ± 5.2 ms). The PDFF quantification shows a slight PDFF bias in the readout direction which leads to an overestimation in the area below the liver dome. The use of the proposed phase correction removes most of the PDFF bias and leads to PDFF values closer to the BH reference scan. This observation also holds true for tissue containing a higher amount of fat as seen in Figure S5. *T*_2_* maps appear mostly unaffected by the phase corrections. Echo images and PDFF maps show visible striation artifacts along the feet‐head direction.

**FIGURE 8:**
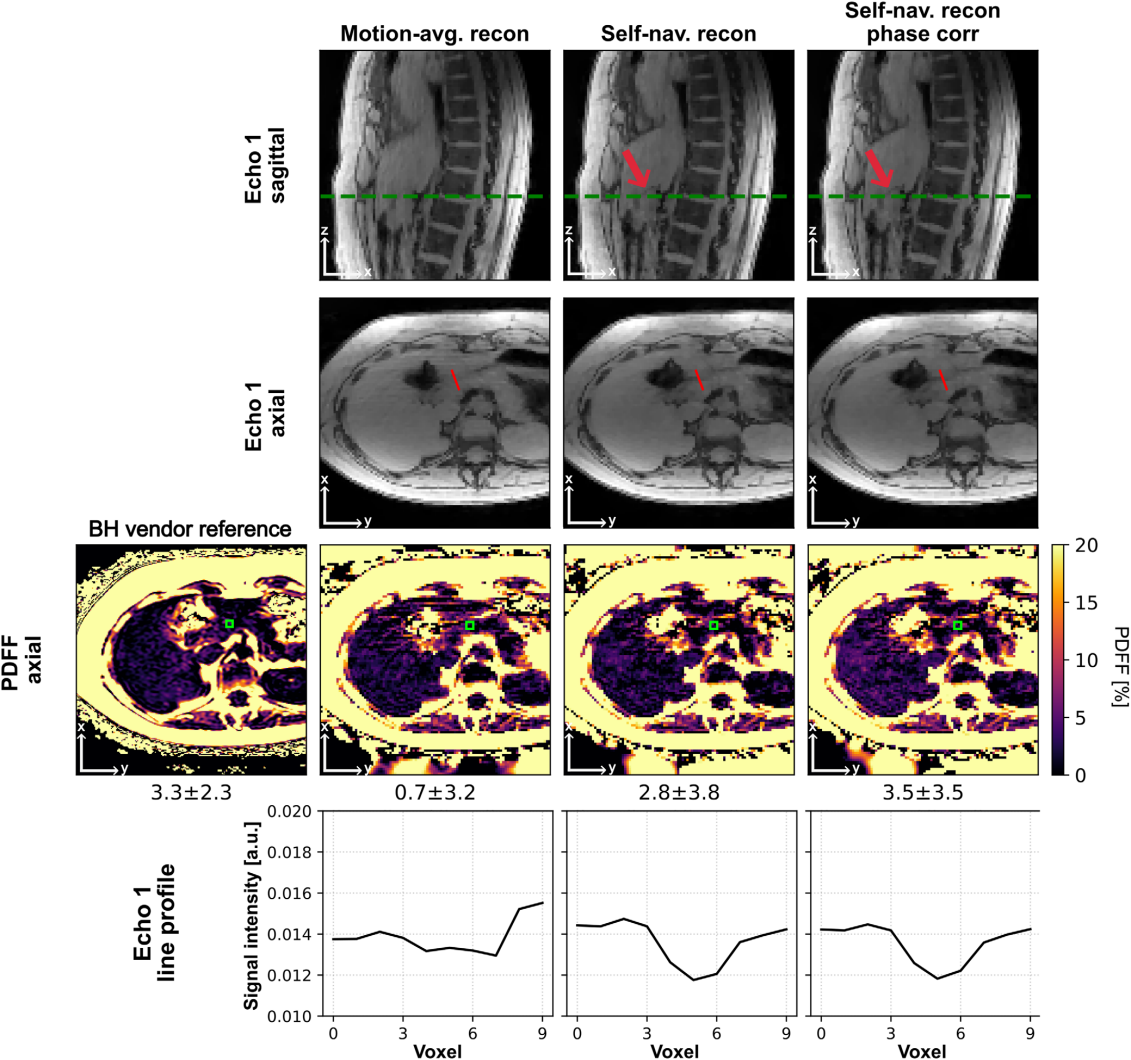
Pancreas first echo magnitude images for a sagital slice as well as first echo magnitude image and PDFF maps for an axial slice along the dashed line (FOV 250 × 400 × 250 mm^3^). From left to right: BH reference scan, motion‐averaged reconstruction, self‐navigated reconstruction, and self‐navigated reconstruction with the proposed linear and concomitant phase correction steps. Red arrows indicate a delineation of the pancreas organ to the surrounding liver organ which appear blurred when motion is not corrected. 1D intensity profiles are plotted along the red line drawn in the echo 1 axial image. After motion correction the boundary to the surrounding organs is clearly visible. PDFF quantification yields similar values to the BH vendor reference scan. Striation patterns are visible in the echo 1 image.

Figure 8 shows an image through a pancreas cross‐section. The effects of the self‐navigated reconstruction and the phase corrections are shown and PDFF maps are compared to a BH reference scan. The pancreas delineation is clearly visible in the echo images and the 1D intensity profile. PDFF quantification inside the exemplary ROI is similar to the BH reference scan. Striation artifacts are visible throughout the image. Figure S6 shows a systematic analysis of the pancreatic delineation across a chosen 1D line profile for all five volunteers. Across all volunteers, the self‐navigated, phase error‐corrected TIMGRECASPR reconstruction exhibits the highest slope, indicating a superior pancreatic delineation when compared to the reference 2 × 3 × 6 mm^3^ Cartesian and the motion‐averaged TIMGRECASPR reconstruction. Based on median results, the self‐navigated TIMGRECASPR sequence demonstrated a 1.5‐fold increase in the measured slope for the 1D profile compared to the motion‐averaged reconstruction, and a 2.9‐fold increase in the measured slope for the 1D profile compared to the Cartesian BH reference scan.

Table 2 shows the measured PDFF values across all five volunteers of a whole liver segmentation obtained from the VibeSegmentator for the BH reference scan, the self‐navigated reconstruction, and the self‐navigated and phase error‐corrected reconstruction. The segmentations are visualized in Figure S2. All three scans performed during the same scan session show similar quantification results. The mean absolute bias across all five volunteers shows a smaller average error for phase‐corrected images at around 0.6%, whereas PDFF without phase corrections is overestimated by around 1.0% on average compared to the reference Cartesian scan. The wSD shows a high repeatability of PDFF quantification results (no phase correction: wSD = 0.07%; with phase correction: wSD = 0.11%). After splitting the segmentation into five different equally thick axial sections, one can observe a significant decrease of the PDFF estimation at the upper liver lobe (Table S1). In this Table, subvolume 5 has been omitted due to a comparably much smaller amount of imaging voxels leading to larger variations. A slight overestimation of the lower liver lobe is seen after performing the proposed phase corrections. An extended analysis of the T2* data inside the liver volume can be found in the Tables S2 and S3. Following the application of the self‐navigated reconstruction and proposed phase corrections, the mean absolute bias for T2* quantification across all five volunteers was 1.2 ms (wSD = 0.48 ms). No significant spatial differences were observed among different liver subvolumes.

**TABLE 2:** Whole liver segmentation PDFF results for five volunteers using the BH reference scan, the self‐navigated six‐echo TIMGRECASPR reconstruction and a self‐navigated six‐echo TIMGRECASPR reconstruction with the proposed phase corrections.

Whole liver PDFF	Scan 1	Scan 2	Scan 3	Mean bias (PDFF %)
Volunteer 1				
BH reference	2.1% ± 3.0%			
Self‐nav. recon	3.1% ± 4.8%	3.0% ± 3.9%	3.1% ± 3.8%	1.0%
Self‐nav. recon, phase corr	2.4% ± 4.6%	2.1% ± 4.0%	2.4% ± 3.9%	0.2%
Volunteer 2				
BH reference	3.0% ± 6.0%			
Self‐nav. recon	4.0% ± 12.9%	4.0% ± 7.6%	3.9% ± 7.1%	1.0%
Self‐nav. recon, phase corr	3.5% ± 7.2%	3.4% ± 7.2%	3.6% ± 7.0%	0.5%
Volunteer 3				
BH reference	3.0% ± 7.0%			
Self‐nav. recon	3.9% ± 8.4%	3.7% ± 8.8%	3.7% ± 8.3%	0.8%
Self‐nav. recon, phase corr	3.7% ± 8.5%	3.6% ± 9.3%	3.5% ± 7.6%	0.6%
Volunteer 4				
BH reference	2.0% ± 4.2%			
Self‐nav. recon	3.2% ± 8.4%	3.1% ± 7.8%	3.1% ± 5.9%	1.1%
Self‐nav. recon, phase corr	2.8% ± 6.1%	2.6% ± 7.5%	2.7% ± 6.2%	0.7%
Volunteer 5				
BH reference	2.1% ± 4.7%			
Self‐nav. recon	3.1% ± 6.1%	3.0% ± 7.5%	3.0% ± 6.8%	0.9%
Self‐nav. recon, phase corr	2.9% ± 6.1%	2.9% ± 6.7%	2.8% ± 6.9%	0.8%

*Note:* The TIMGRECASPR scan was performed three times to check for short‐term variability. The mean absolute bias was calculated relative to the BH reference scan. Across all volunteers, the mean absolute PDFF bias was 1.0% with wSD = 0.07% for the self‐navigated reconstruction without the proposed phase corrections. The mean absolute PDFF bias was 0.6% with wSD = 0.11% for the self‐navigated reconstruction with the proposed phase corrections.

Measured PDFF values within the whole pancreas segmentation for both the reference Cartesian scan and the self‐navigated, phase error‐corrected TIMGRECASPR reconstruction are presented in Table S4. Across all three repeated scans, the measured PDFF values show similar and repeatable quantification results, yielding a mean absolute bias of 1.1% PDFF compared to the reference Cartesian scan (wSD = 0.37%). Except for volunteer 1, the proposed TIMGRECASPR sequence slightly overestimates the pancreatic PDFF compared to the reference Cartesian scan.

Pearson *R* ^2^ demonstrates a strong correlation between whole liver and pancreas mean PDFF measurements from the reference Cartesian and TIMGRECASPR scans for all five volunteers (Figure S7). The Bland–Altman analysis highlights a slight bias, with TIMGRECASPR acquisitions generally yielding slightly higher PDFF values than the reference Cartesian scans. In vivo PDFF maps utilizing a broader 0%–100% PDFF color scale are provided in the Figures S8–S10. PDFF quantification within fatty tissue demonstrates comparable quality to that of the reference Cartesian scan.

## 4 Discussion

A novel approach for free‐breathing PDFF quantification was developed which uses a monopolar time‐interleaved CASPR trajectory instead of the commonly used SoS. Both trajectories can make use of their inherent self‐navigation capabilities due to the oversampling of the k‐space center in each shot. This enables retrospective motion correction without the need for any additional motion navigators or hardware [28, 30]. The CASPR trajectory enables high isotropic spatial resolution for PDFF quantification compared to current clinical protocols.

Free‐breathing fat quantification in the abdomen is primarily confounded by two sources of error: eddy currents and motion artifacts: eddy currents and motion artifacts. Eddy currents can severely affect quantification results especially when a complex based water‐fat separation technique is used. Complex‐based water‐fat separation techniques are preferred for quantitative PDFF imaging due to their superior noise performance and robustness to fat modeling errors [45, 46]. However, these techniques are more susceptible to phase errors. The phase corrections applied in this work assume that there are two dominant contributors to phase errors. Linear phase errors $\Phi_{\mathrm{lin}}$ originating from eddy currents in readout direction and phase errors due to concomitant gradients $\Phi_{\text{concom}}$. A phase offset between interleaves was also proposed by Ruschke et al. [52], but its influence was considered negligible for the echo‐time steps used in this work. The efficacy and relevancy of the assumed phase error corrections were first tested in phantoms and a non‐moving anatomy (thigh) before their application to a moving anatomy (liver).

Beyond phase errors caused by eddy currents, physiological motion represents the second major challenge, leading not only to blurred images but also to quantification errors. To motivate the use of the TIMGRECASPR trajectory for free‐breathing high isotropic resolution of the abdomen, a simulation using the XCAT phantom [61] with different PE_shot_ was performed. Increasing PE_shot_ also increases the shot length, which affects the ability to perform self‐navigation and averaging motion effects over the whole shot duration. The simulation results demonstrate that only a soft‐gated reconstruction using PE_shot_ = 25 was able to fully resolve the whole breathing curve and show maps where the pancreas was clearly delineated from surrounding organs. These maps looked similar to a simulation where no inherent motion was applied to the XCAT phantom. However, the effective undersampling might differ between scans using different PE_shot_ and constant scan time, potentially leading to remaining artifacts especially for very low or high PE_shot_, which can lead to certain undersampling patterns (see PSFs in Figure S1). For lower PE_shot_, eddy currents could also play a more significant role due to the larger gradient jumps between phase‐encoding steps.

As the phase errors depend on the chosen echo time combination and the extent of the FOV, a high resolution scan of a large water phantom was obtained to highlight the PDFF bias on a true 0% PDFF caused by gradient imperfections. Even though the scan was acquired using a monopolar TIMGRE acquisition, a correction of the generated linear phase error was still required. This is likely due to echo‐specific phase errors, which then result in erroneous water‐fat quantification for chemical shift encoding‐based techniques, since they rely on multiple echoes to fit a modeled signal evolution over echo time. The remaining errors in the periphery of the phantom were successfully mitigated using the concomitant gradient correction leading to homogeneous PDFF maps throughout the large FOV. Some errors remain, which are potentially caused by eddy currents from the phase‐encoding gradients along the spiral dimension or by higher order effects which are not accounted for here.

PDFF maps of the water‐fat phantom show only minor phase‐related quantification errors. Moreover, the linear phase correction is unable to remove the whole linear bias in the water background. However, errors remain low and the PDFF quantification inside the fat vials shows improved results in the Bland–Altmann plots compared to a previous fully sampled high resolution Cartesian TIMGRE scan. These remaining errors might be due to a buildup of an additional phase error due to the sampling of the k‐space center at the middle of the shot during the used spiral‐in/out trajectory. This error, however, is small, and after the additional concomitant correction, we obtain a mostly homogeneous water background and accurate quantification of the PDFF inside the fat vials.

Moving on to an in vivo anatomy, two thigh scans of the same volunteer with varying echo time step were evaluated. Phase errors are mostly visible on the bottom high resolution thigh image where the echo time step becomes suboptimal for water‐fat separation. The phase corrections had the intended effect and the underestimation of the PDFF at *z* = 75 mm is successfully mitigated. The concomitant phase correction can correct for PDFF underestimation at the periphery of the thigh. Its effect was varying with the x‐y distance from the isocenter, as confirmed by our previous XCAT simulation. For the low‐resolution thigh image, artifacts due to phase errors are small; nonetheless, a linear phase correction was needed to correct for the occurring linear phase.

After verification of the proposed framework the reconstruction pipeline was applied to the abdomen where we also utilize the self‐navigating capabilities of the trajectory. Significant blurring of the echo images and resulting quantification errors for both PDFF as well as *T*_2_* are visible throughout the whole liver volume when a motion‐averaged reconstruction was used. Ghosting artifacts from motion are clearly visible in the liver volume. Using a self‐navigated reconstruction provides much sharper echo images with clearly defined boundaries between the liver dome and the lungs. *T*_2_* maps—an imaging parameter highly susceptible to remaining motion artifacts—appeared sharper. However, PDFF estimation inside the given ROIs shows a clear overestimation of the PDFF inside the liver volume mostly visible toward the liver dome. This effect was successfully mitigated by incorporating both the linear and the concomitant phase corrections. Values became more consistent to the BH reference scan. *T*_2_* appears largely unaffected by the phase corrections and was mostly affected by the inclusion of the self‐navigated reconstruction. TIMGRECASPR PDFF quantification results appear stable throughout the whole segmented liver volume for repeated scans and for different volunteers. For all volunteers, the mean absolute bias of the phase corrected image is smaller when applying the proposed phase corrections with values closely matching those of the BH reference scan. A slight overestimation of the liver PDFF in the lower liver lobe is observed, which may be due to remaining linear phase errors in the image.

In agreement with our XCAT simulation predictions, the in vivo evaluations demonstrated that utilizing short shot lengths of PE_shot_ = 25 in combination with motion navigation provides improved delineation of the pancreas from surrounding organs. The visual assessment was quantitatively corroborated by systematic 1D line profile analyses. Across all subjects, the self‐navigated, phase error‐corrected TIMGRECASPR reconstruction yielded the steepest slopes across the pancreatic boundary—indicating higher edge sharpness—than both the simple motion‐averaged reconstruction and the reference Cartesian scan. The difficulty in boundary delineation observed with the reference Cartesian scan is largely attributable to its 6 mm slice thickness, which introduces substantial partial volume effects. These effects severely complicate evaluation in sagittal reformats and blur the pancreatic outline even in standard axial planes. In contrast, the proposed self‐navigated TIMGRECASPR reconstruction enables high isotropic spatial resolution and volumetric coverage necessary for precise organ definition. PDFF quantification across the entire pancreatic volume demonstrates low intra‐scan variability and good correlation with measurements from the reference Cartesian scan. These results show that the self‐navigated, phase error‐corrected TIMGRECASPR sequence effectively mitigates motion artifacts, which otherwise lead to blurred pancreatic boundaries and quantification errors. However, it is important to note that the DICE similarity coefficients reported for pancreas segmentation using the VibeSegmentator are only moderate (0.73–0.81). This can influence quantification results and potentially lead to higher standard deviations if adjacent visceral fat is inadvertently included. Despite this limitation, Pearson R^2^ demonstrated a strong correlation between PDFF measurements derived from the proposed self‐navigated, phase error‐corrected TIMGRECASPR sequence and the reference BH Cartesian scan across various tissues with differing fat content.

Various different chemical shift encoding‐based methods for free‐breathing PDFF quantification exist such as radial SoS sequences [22, 25, 28, 30, 31, 32, 33, 63] or 2D sequential techniques [26, 27]. Three main benefits can be observed using the TIMGRECASPR trajectory. First, the TIMGRECASPR trajectory allows to easily adapt the shot length to keep a high temporal resolution of the motion self‐navigation. Second, compared to non‐Cartesian trajectories, the TIMGRECASPR has a lower sensitivity to gradient errors, which are also more straightforward to correct due to the readout always being in the same direction. Third, TIMGRECASPR acquisitions benefit from shorter reconstruction times as it does not require a non‐uniform Fourier transform in contrast to other non‐Cartesian MRI methods.

The proposed method presents some limitations. First, the concomitant phase error correction may be affected by higher order terms arising from phase‐encoding gradients due to the use of a spiral ordering in the phase‐encoding dimension. This might cause the remaining visible PDFF artifacts and residual bias in the phantoms and in vivo. Second, since CASPR samples the k‐space center later in the shot rather than at the beginning, eddy currents from the phase‐encoding gradients might impact the acquisition of the k‐space center. Using this spiral‐in/out sampling could also potentially affect the quality of the linear phase error correction, where the calibration lines used to calculate the offset have been acquired during a steady state. Third, the current protocol acquires the calibration lines in a short BH to eliminate potential motion artifacts. Further testing is needed whether this BH is essential as the acquisition of this one‐dimensional calibration line in both polarities is very fast compared to the breathing frequency. Fourth, while the soft‐gated reconstruction achieves satisfactory results and accurate PDFF and *T*_2_* quantification, there are remaining striation artifacts visible in the images. These potentially stem from motion or undersampling artifacts and lead to small local inhomogeneities of the PDFF image. A more thorough tuning of parameters of the self‐navigated motion gating may improve motion robustness. Moreover, the acquisition scheme of the trajectory in this work can be optimized. Using a very low number of PE_shot_ may lead to structured ring‐like undersampling artifacts in the PSF and a higher susceptibility to eddy current artifacts occurring between the larger gradient jumps in the phase‐encoding dimension. Using very high PE_shot_, however, can lead to incoherent undersampling artifacts in the PSF after motion self‐navigation and limits its temporal resolution. A careful tuning of the PE_shot_ number is required depending on the application. Finally, a broader validation in a larger cohort of patients remains necessary. The technique and the proposed phase corrections need to be further evaluated in a cohort of patients with hepatic and pancreatic steatosis both in terms of PDFF and T2* mapping in the presence of elevated PDFF values. Moreover, the pancreas in young people or the XCAT phantom may look very different when compared to older people. Further validation needs to be performed on an older population whether their pancreas can still be resolved clearly after the proposed soft‐gated reconstruction.

## 5 Conclusion

A novel monopolar time‐interleaved six‐echo CASPR acquisition is feasible for free breathing quantitative PDFF mapping when linear and concomitant phase errors are accounted for. The self‐navigation capability of the CASPR trajectory enables it to be used also for motion‐affected anatomies such as the liver. Due to the isotropic resolution combined with short shot lengths for motion self‐navigation, small organs such as the pancreas can be resolved clearly.

## Funding

This work was supported by Deutsche Forschungsgemeinschaft (455422993/FOR5298‐iMAGO‐P1).

## Conflicts of Interest

Johannes M. Peeters is an employee of Philips Healthcare, Holger Eggers is an employee of Philips GmbH Innovative Technologies. Kilian Weiss is an employee of Philips GmbH Market DACH. Dimitrios Karampinos received grant support from Philips Healthcare while at the Technical University of Munich.

## Supporting information


**Figure S1:** Representative spiral‐in/out phase‐encoding trajectories for varying PE_shot_ and the resulting PSF for all three simulated trajectories as well as a PE_shot_ of 10. PE_shot_ denotes the number of phase encoding sampling points per spiral shot. PSFs are computed for both the fully sampled scan and the retrospectively undersampled k‐space after applying self‐navigated soft‐gating weights for motion compensation (without applying any regularization). These weights are derived from the motion curves shown in Figure S3 for both cases. No regularization is applied in the reconstruction. To maintain a constant total scan time across all three simulated sampling schemes, an increased PE_shot_ is compensated by acquiring fewer spiral interleaves in total. A total of 1543, 617, 309, or 154 phase‐encoding spirals were acquired for the different PE_shot_ numbers, respectively. The PSF exhibits strong ring‐like undersampling artifacts for a very low PE_shot_ of 10 where no sufficient coverage in the $k_{y}-k_{z}$ space can be ensured. For larger numbers of PE_shot_, such ring‐like undersampling artifacts have significantly reduced energy in the PSF. Ring‐like undersampling artifacts with much reduced energy can be observed in the PSF for PE_shot_ numbers of 50 and 100 which get considerable worse following the application of motion self‐navigation due to the effectively stronger total undersampling.
**Figure S2:** Whole liver segmentations used in Table 2. The liver segmentations, overlaid on the echo image in red, were created using the TotalVibeSegmentator on a previously reconstructed water image (reported DICE coefficients for liver segmentations 0.93–0.94). For each volunteer the liver segmentation volume is split into five different axial subvolumes, which are equally thick as illustrated here in cyan. These subvolumes are analyzed separately in Table S2 to assess the spatial homegeneity of the liver PDFF across the feet/head liver dimension. Small under‐ and oversegmentation errors can be observed.
**Figure S3:** PCA‐derived motion curves from the XCAT simulation incorporating a 5 s respiratory cycle. The acquisitions were simulated using a PE_shot_ of 25, 50, and 100 (corresponding to shot lengths of 311, 622, and 1244 ms respectively). While a PE_shot_ of 25 provides sufficient temporal resolution to accurately capture the full respiratory cycle, a higher PE_shot_ fails to adequately resolve the breathing motion in the temporal domain.
**Figure S4:** Simulated PDFF bias for a 0% PDFF reference due to phase errors induced by concomitant gradient fields from gradient echo trains (FOV 250 × 250 × 400 mm^3^). Rows correspond to different echo time combinations, while columns display different slice orientations. A strong dependence of concomitant gradient‐induced errors on the applied echo time combination can be observed. These errors manifest primarily as concentric rings centered at the magnet isocenter in the scanner *x*–*y* plane. For in vivo experiments, the echo time combinations from the first and last rows were used for thigh imaging, while only the combination from the first row was used for abdominal imaging.
**Figure S5:** Regression and Bland–Altman analysis for whole liver and pancreas mean PDFF measurements presented in Table 2 and Table S4 as well as ROIs drawn in subcutaneous tissue, spleen and vertebral bone marrow. The Bland–Altman plot displays the measurement differences, calculated from the Cartesian BH minus the TIMGRECASPR scan. Across the whole fat fraction range, the squared Pearson correlation coefficient (*R* ^2^) demonstrates a strong correlation between mean PDFF values from the reference Cartesian BH and the TIMGRECASPR scan. However, a slight bias is observed, with TIMGRECASPR generally yielding slightly higher PDFF values than the reference Cartesian BH.
**Figure S6:** Sagittal and axial water images of a pancreatic cross‐section for all five volunteers. The green contour highlights the pancreatic outline generated from the VibeSegmentator. 1D line profiles are drawn across the pancreatic boundary. A line is fitted to the central portion of the profile, corresponding to the presumed boundary location. The resulting slope represents edge sharpness, serving as an index of pancreatic delineation along the given line profile. Consistently across all volunteers, the self‐navigated TIMGRECASPR reconstruction exhibits the highest slope. This suggests superior delineation of the pancreas from surrounding tissue compared to both a 2 × 3 × 6 mm^3^ breath‐hold (BH) Cartesian acquisition and a motion‐averaged TIMGRECASPR reconstruction.
**Figure S7:** Regression and Bland–Altman analysis for whole liver and pancreas mean PDFF measurements presented in Table 2 and Table S4. The Bland–Altman plot displays the measurement differences, calculated from the Cartesian BH minus the free‐breathing TIMGRECASPR scan. The squared Pearson correlation coefficient (*R* ^2^) demonstrates a strong correlation between mean PDFF values from the reference Cartesian BH and the TIMGRECASPR scan. However, a slight bias is observed, with TIMGRECASPR generally yielding slightly higher PDFF values than the reference Cartesian BH.
**Figure S8:** Figure 6 with a wider 0%–100% PDFF scale.
**Figure S9:** Figure 7 with a wider 0%–100% PDFF scale.
**Figure S10:** Figure 8 with a wider 0%–100% PDFF scale.
**Table S1:** Mean PDFF of the whole liver segmentation and different axial subvolumes splitting the segmentation from Figure S1 into five equally thick axial volumes. Subvolume 5 has been omitted from this table due to a comparably much lower number of imaging voxels. Generally, the TIMGRECASPR method is slightly overestimating the liver PDFF compared to the Cartesian BH reference scan. The phase correction successfully corrects the overestimation at the upper liver lobe. However, in many subjects it leads to a slight overestimation of the lower liver lobe. This only contributes to a lesser extent to the total mean PDFF due to its smaller volume.
**Table S2:** Whole liver segmentation T2* results for five volunteers using the BH reference scan and a self‐navigated six echo TIMGRECASPR reconstruction with the proposed phase corrections. The TIMGRECASPR scan was performed three times to check for short‐term variability. The mean bias was calculated relative to the BH reference scan. Across all volunteers, the mean absolute T2* bias was 1.2 ms with wSD = 0.48 ms.
**Table S3:** Mean *T*_2_* of the whole liver segmentation and different axial subvolumes splitting the segmentation from Figure S1 into five equally thick axial volumes. Subvolume 5 has been omitted from this table due to a comparably much lower number of imaging voxels. Generally, agreement between the TIMGRECASPR and the Cartesian BH reference scan is quite well throughout the whole imaging volume. Standard deviations of the *T*_2_* do not differ significantly throughout the subvolumes. This means that the proposed motion self‐navigation has been effective in reducing motion artifacts particularly at the upper liver lobe.
**Table S4:** Whole pancreas segmentation PDFF results for five volunteers using the BH reference scan and a self‐navigated six echo TIMGRECASPR reconstruction with the proposed phase corrections. Pancreatic segmentations were obtained from the TotalVibeSegmentator on a previously reconstructed water image (reported DICE coefficients for pancreatic segmentations 0.73–0.81). The TIMGRECASPR scan was performed three times to check for short‐term variability. The mean bias was calculated relative to the BH reference scan. Across all volunteers, the mean absolute PDFF bias was 1.1% with wSD = 0.37%.

## Data Availability

The data that support the findings of this study are available on request from the corresponding author. The data are not publicly available due to privacy or ethical restrictions.
