# Supplementary material for: Free‐Breathing Fat Quantification Using a Phase Error‐Corrected Cartesian Acquisition With Spiral Profile Ordering
Source: Magn Reson Med. 2026 Jun 16;96(4):1709–25. doi: 10.1002/mrm.70474 (PMC13418965; doi:10.1002/mrm.70474)
Supplement: Supplementary file 1 — Figure S1: Representative spiral‐in/out phase‐encoding trajectories for varying PEshot and the resulting PSF for all three simulated trajectories as well as a PEshot of 10. PEshot denotes the number of phase encoding sampling points per spiral shot. PSFs are computed for both the fully sampled scan and the retrospectively undersampled k‐space after applying self‐navigated soft‐gating weights for motion compensation (without applying any regularization). These weights are derived from the motion curves shown in Figure S3 for both cases. No regularization is applied in the reconstruction. To maintain a constant total scan time across all three simulated sampling schemes, an increased PEshot is compensated by acquiring fewer spiral interleaves in total. A total of 1543, 617, 309, or 154 phase‐encoding spirals were acquired for the different PEshot numbers, respectively. The PSF exhibits strong ring‐like undersampling artifacts for a very low PEshot of 10 where no sufficient coverage in the ky−kz space can be ensured. For larger numbers of PEshot, such ring‐like undersampling artifacts have significantly reduced energy in the PSF. Ring‐like undersampling artifacts with much reduced energy can be observed in the PSF for PEshot numbers of 50 and 100 which get considerable worse following the application of motion self‐navigation due to the effectively stronger total undersampling. Figure S2: Whole liver segmentations used in Table 2. The liver segmentations, overlaid on the echo image in red, were created using the TotalVibeSegmentator on a previously reconstructed water image (reported DICE coefficients for liver segmentations 0.93–0.94). For each volunteer the liver segmentation volume is split into five different axial subvolumes, which are equally thick as illustrated here in cyan. These subvolumes are analyzed separately in Table S2 to assess the spatial homegeneity of the liver PDFF across the feet/head liver dimension. Small under‐ and oversegmentation errors can b [file MRM-96-1709-s001.docx]

**Free-breathing fat quantification using a phase error-corrected Cartesian acquisition with spiral profile ordering (CASPR)**

**Supplementary material**


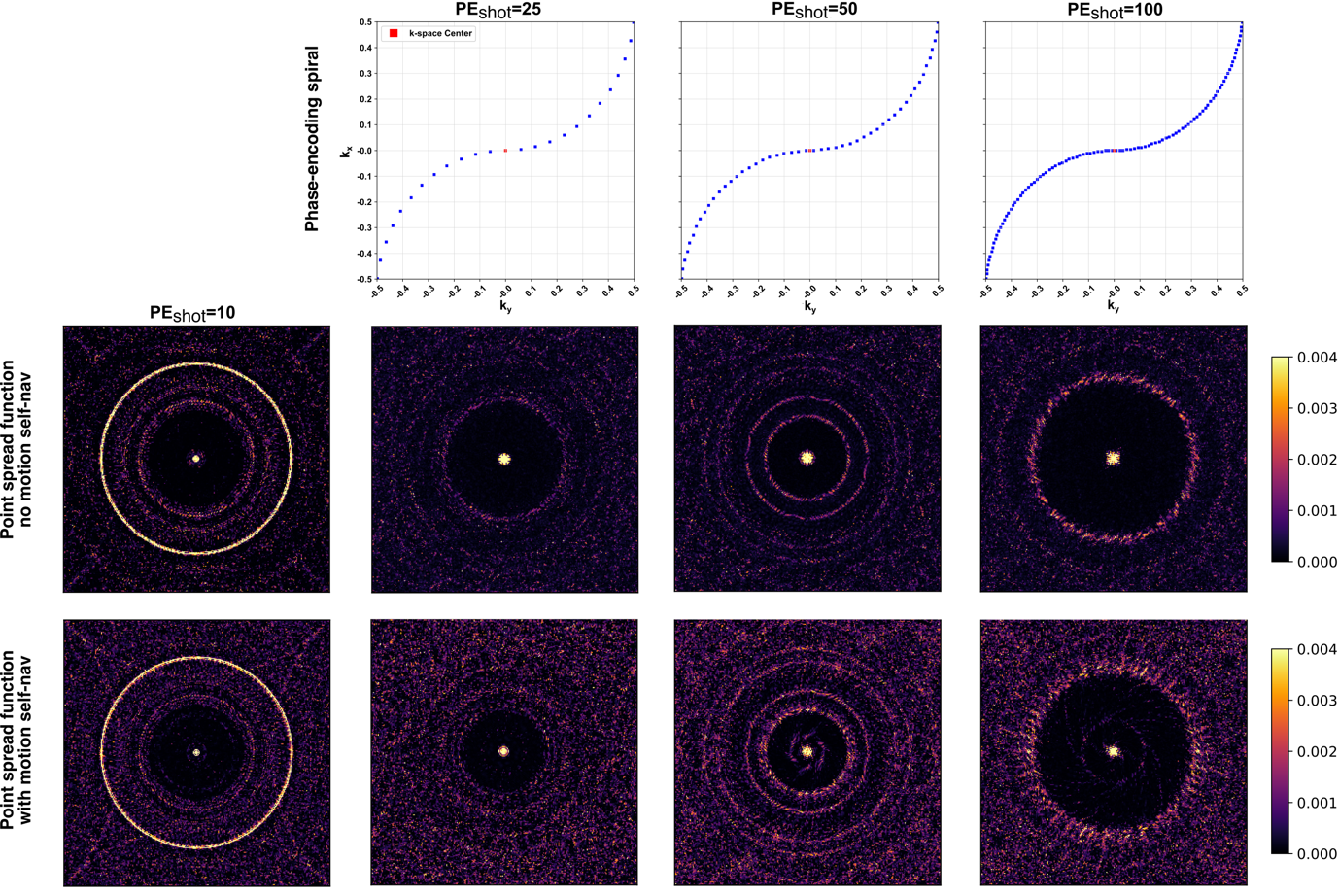


FIG. S1: Representative spiral-in/out phase-encoding trajectories for varying PE_shot_ and the resulting PSF for all three simulated trajectories as well as a PE_shot_ of 10. PE_shot_ denotes the number of phase encoding sampling points per spiral shot. PSFs are computed for both the fully sampled scan and the retrospectively undersampled k-space after applying self-navigated soft-gating weights for motion compensation (without applying any regularization). These weights are derived from the motion curves shown in FIG. S3 for both cases. No regularization is applied in the reconstruction. To maintain a constant total scan time across all three simulated sampling schemes, an increased PE_shot_ is compensated by acquiring fewer spiral interleaves in total. A total of 1543, 617, 309, or 154 phase-encoding spirals were acquired for the different PE_shot_ numbers, respectively. The PSF exhibits strong ring-like undersampling artifacts for a very low PE_shot_ of 10 where no sufficient coverage in the k_y_-k_z_ space can be ensured. For larger numbers of PE_shot_, such ring-like undersampling artifacts have significantly reduced energy in the PSF. Ring-like undersampling artifacts with much reduced energy can be observed in the PSF for PE_shot_ numbers of 50 and 100 which get considerable worse following the application of motion self-navigation due to the effectively stronger total undersampling.


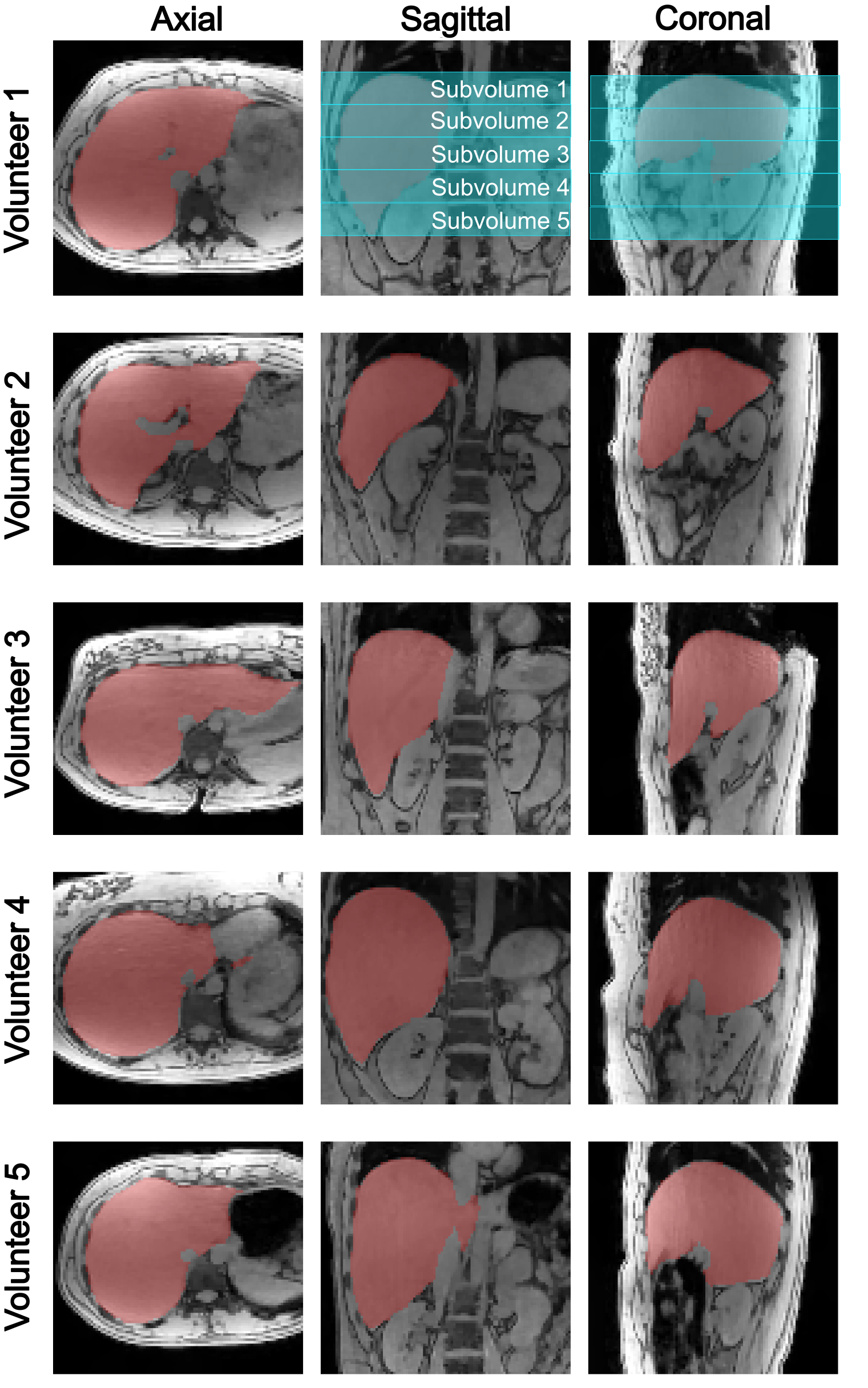


FIG. S2: Whole liver segmentations used in Table 2. The liver segmentations, overlaid on the echo image in red, were created using the TotalVibeSegmentator on a previously reconstructed water image (reported DICE coefficients for liver segmentations 0.93-0.94). For each volunteer the liver segmentation volume is split into five different axial subvolumes, which are equally thick as illustrated here in cyan. These subvolumes are analyzed separately in Table S2 to assess the spatial homegeneity of the liver PDFF across the feet/head liver dimension. Small under- and oversegmentation errors can be observed.


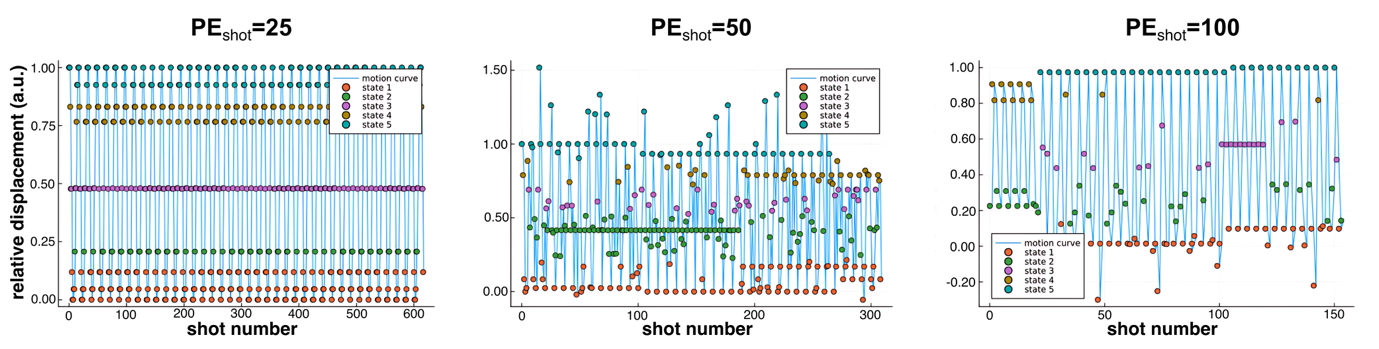


FIG. S3: PCA-derived motion curves from the XCAT simulation incorporating a 5 s respiratory cycle. The acquisitions were simulated using a PE_shot_ of 25, 50 and 100 (corresponding to shot lengths of 311, 622 and 1244 ms respectively). While a PE_shot_ of 25 provides sufficient temporal resolution to accurately capture the full respiratory cycle, a higher PE_shot_ fails to adequately resolve the breathing motion in the temporal domain.
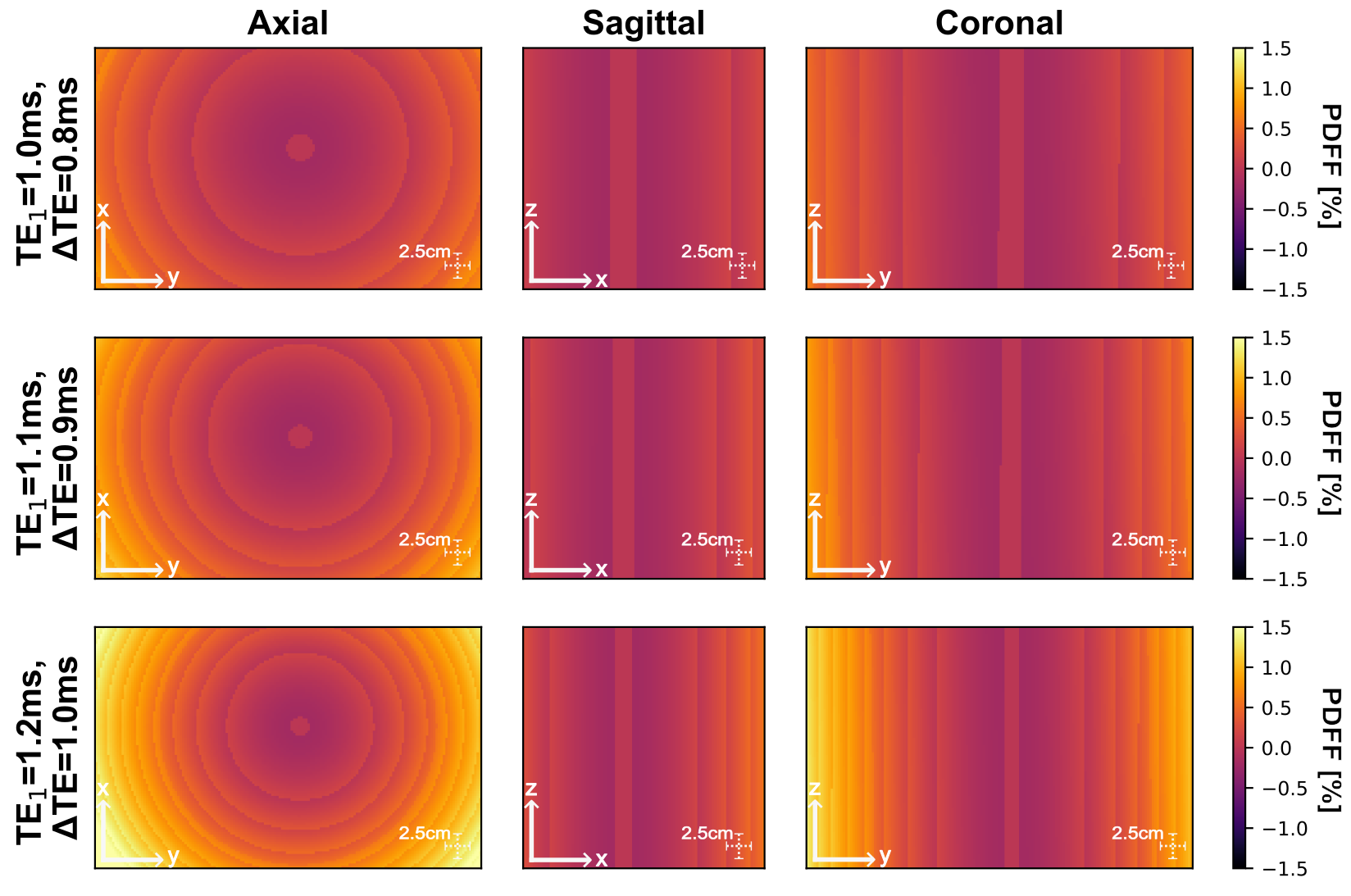


FIG. S4: Simulated PDFF bias for a 0 % PDFF reference due to phase errors induced by concomitant gradient fields from gradient echo trains (FOV 250 x 250 x 400 mm^3^). Rows correspond to different echo time combinations, while columns display different slice orientations. A strong dependence of concomitant gradient-induced errors on the applied echo time combination can be observed. These errors manifest primarily as concentric rings centered at the magnet isocenter in the scanner x-y plane. For in vivo experiments, the echo time combinations from the first and last rows were used for thigh imaging, while only the combination from the first row was used for abdominal imaging.


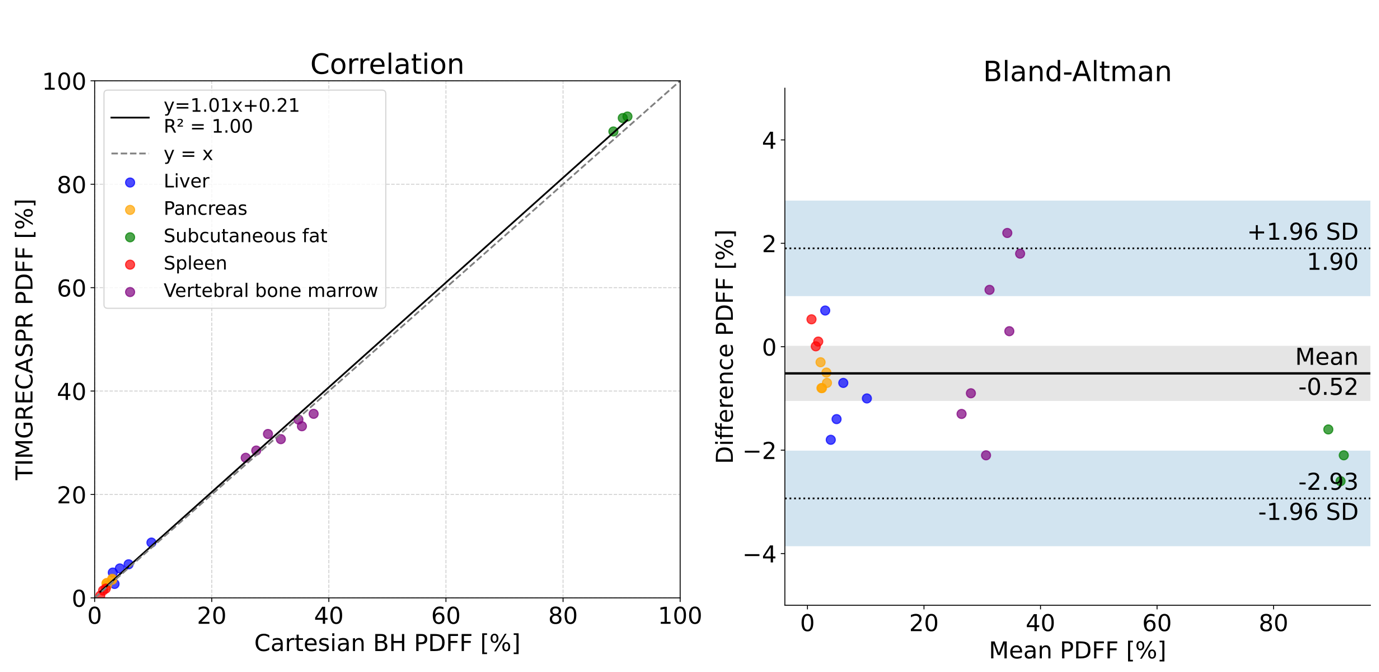


FIG. S5: Regression and Bland-Altman analysis for whole liver and pancreas mean PDFF measurements presented in Table 2 and Table S4 as well as ROIs drawn in subcutaneous tissue, spleen and vertebral bone marrow. The Bland-Altman plot displays the measurement differences, calculated from the Cartesian BH minus the TIMGRECASPR scan. Across the whole fat fraction range, the squared Pearson correlation coefficient (R^2^) demonstrates a strong correlation between mean PDFF values from the reference Cartesian BH and the TIMGRECASPR scan. However, a slight bias is observed, with TIMGRECASPR generally yielding slightly higher PDFF values than the reference Cartesian BH.


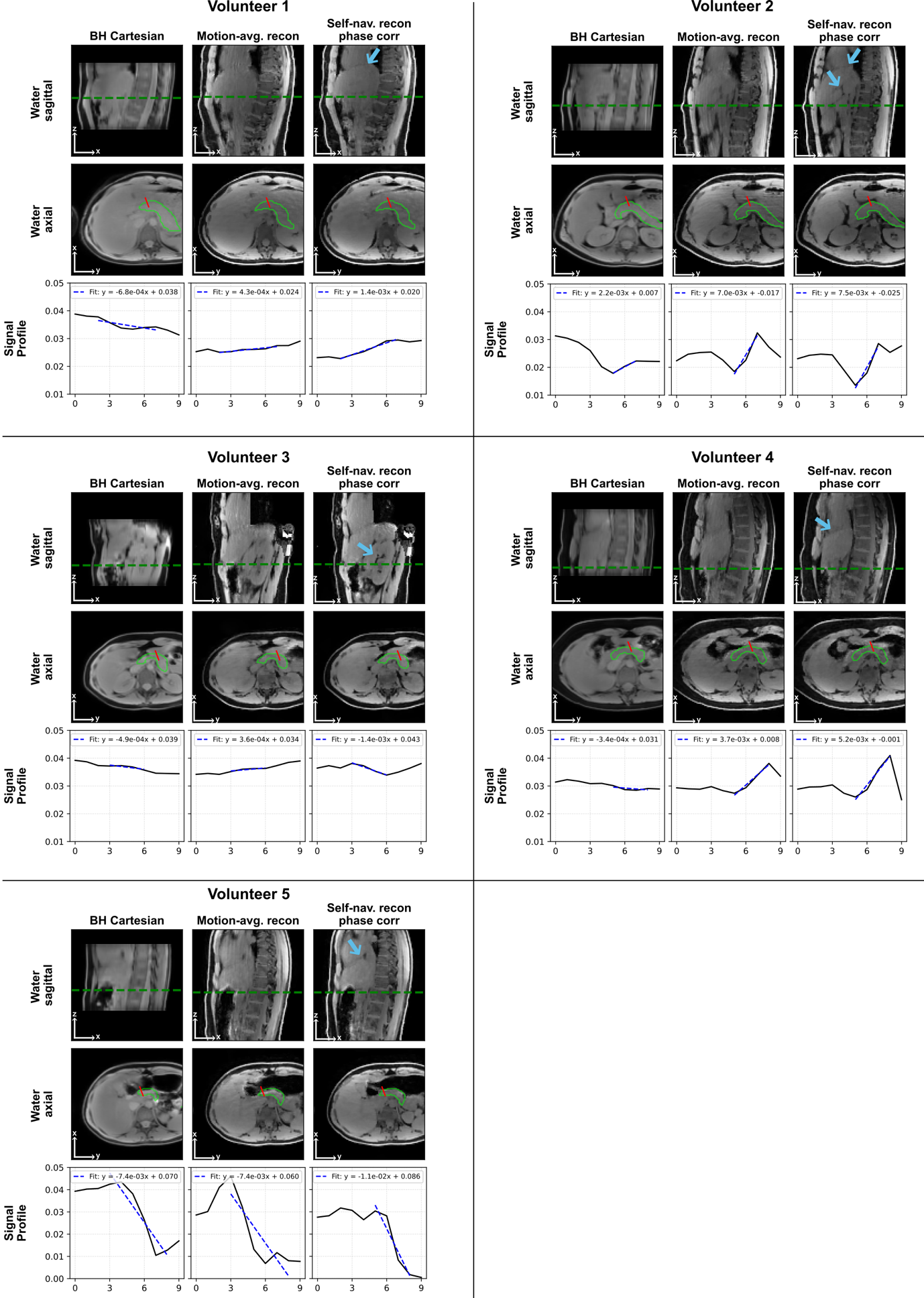


FIG. S6: Sagittal and axial water images of a pancreatic cross-section for all five volunteers. The green contour highlights the pancreatic outline generated from the VibeSegmentator. 1D line profiles are drawn across the pancreatic boundary. A line is fitted to the central portion of the profile, corresponding to the presumed boundary location. The resulting slope represents edge sharpness, serving as an index of pancreatic delineation along the given line profile. Consistently across all volunteers, the self-navigated TIMGRECASPR reconstruction exhibits the highest slope. This suggests superior delineation of the pancreas from surrounding tissue compared to both a 2 × 3 × 6 mm³ breath-hold (BH) Cartesian acquisition and a motion-averaged TIMGRECASPR reconstruction.


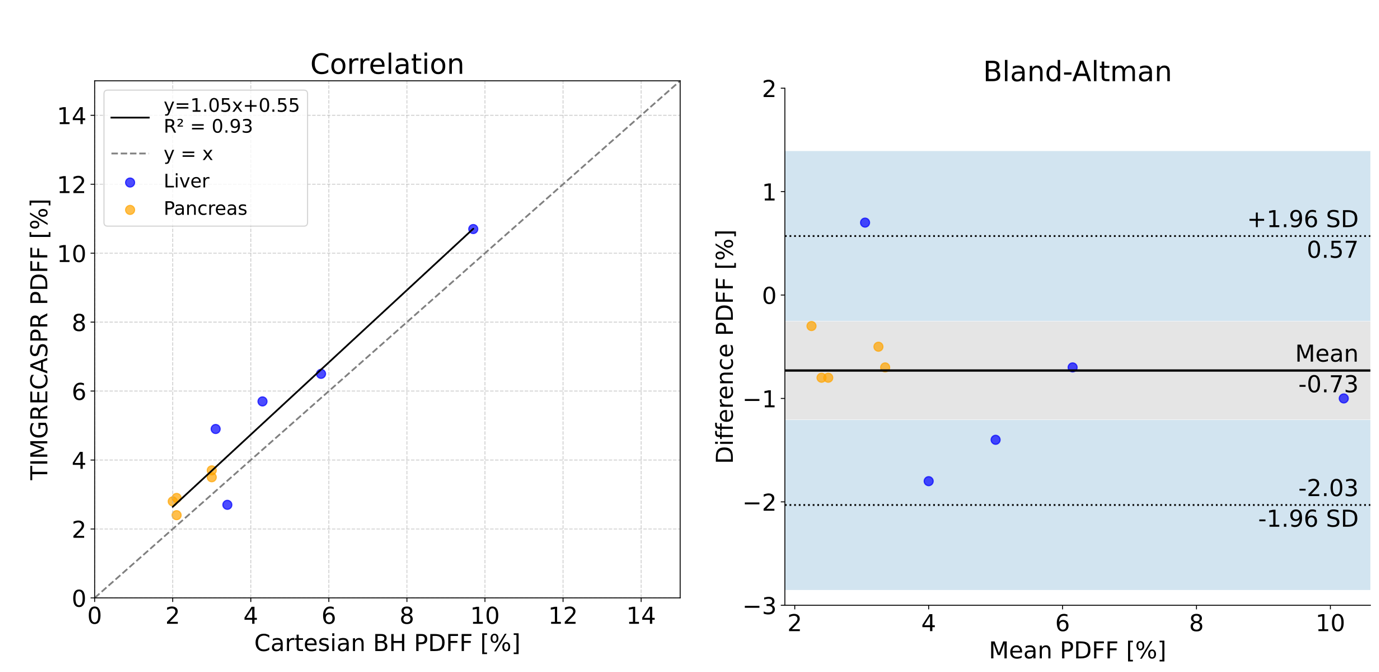


FIG. S7: Regression and Bland-Altman analysis for whole liver and pancreas mean PDFF measurements presented in Table 2 and Table S4. The Bland-Altman plot displays the measurement differences, calculated from the Cartesian BH minus the free-breathing TIMGRECASPR scan. The squared Pearson correlation coefficient (R^2^) demonstrates a strong correlation between mean PDFF values from the reference Cartesian BH and the TIMGRECASPR scan. However, a slight bias is observed, with TIMGRECASPR generally yielding slightly higher PDFF values than the reference Cartesian BH.


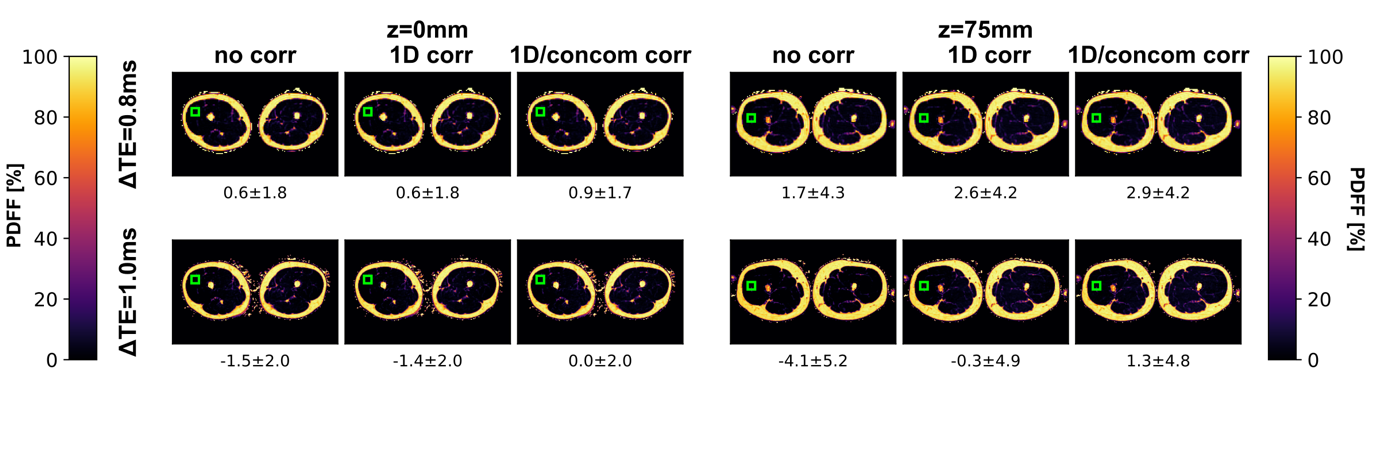
FIG. S8: Figure 6 with a wider 0 to 100 % PDFF scale.


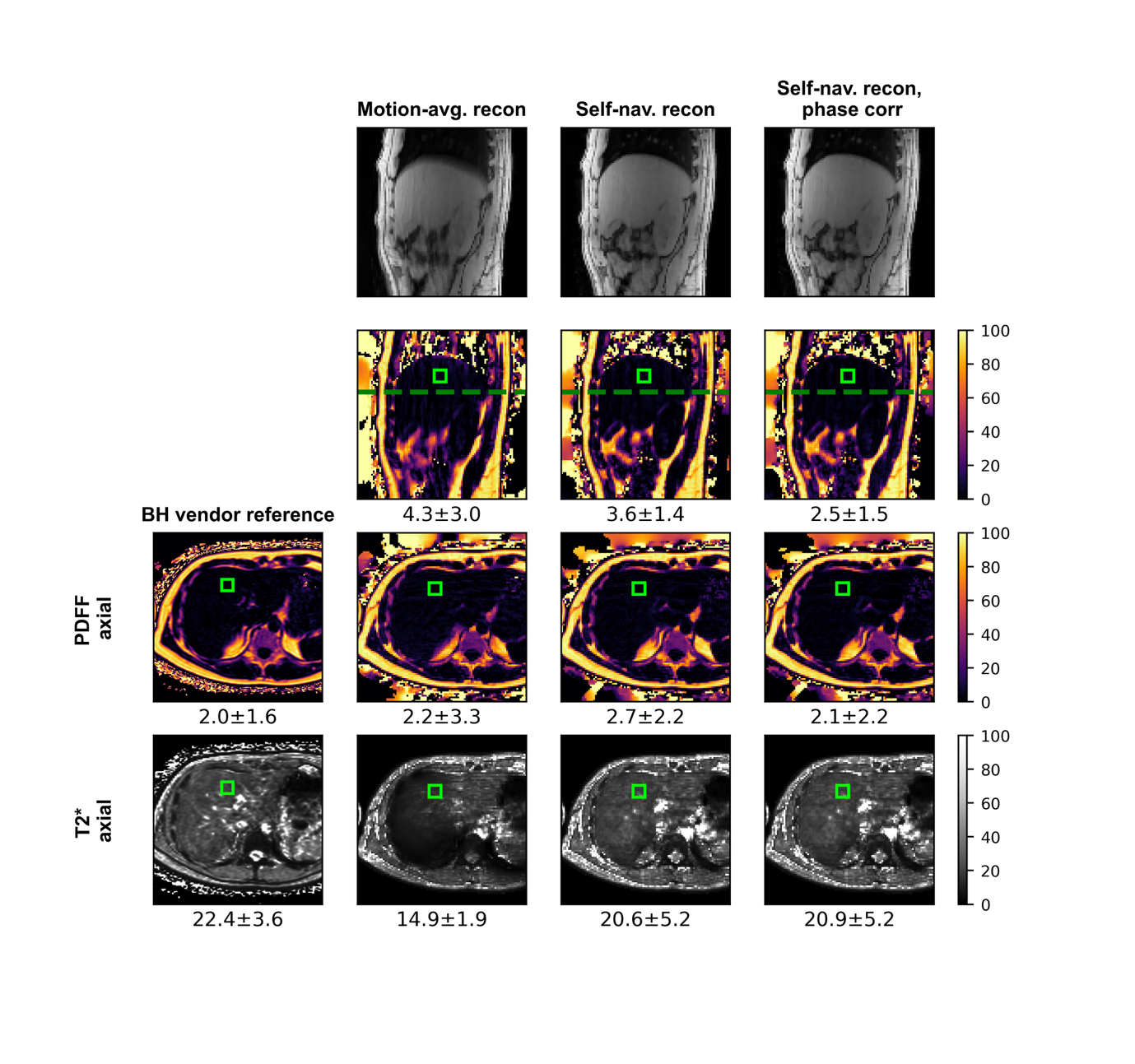


FIG. S9: Figure 7 with a wider 0 to 100 % PDFF scale.


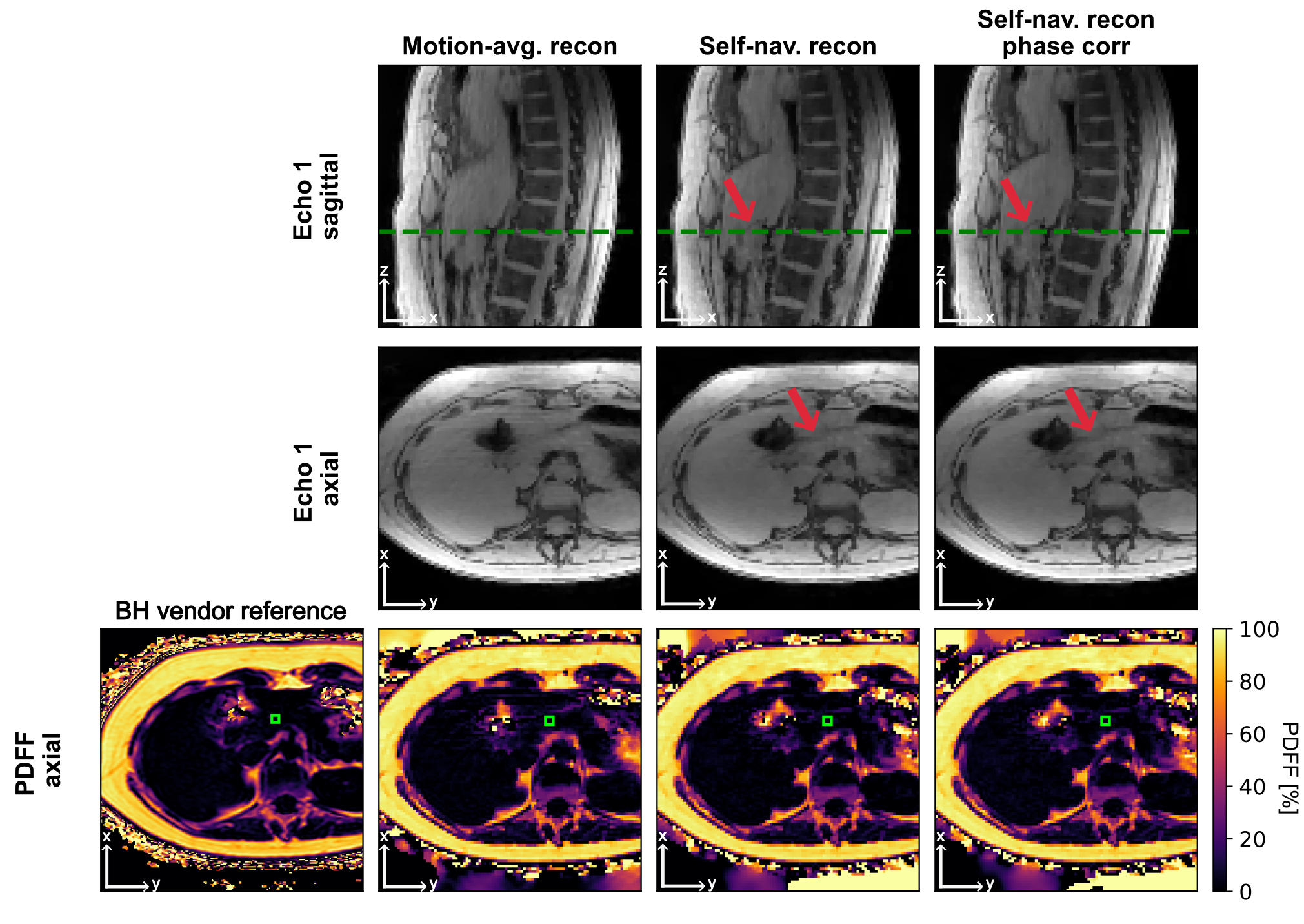


FIG. S10: Figure 8 with a wider 0 to 100 % PDFF scale.

Table S1: Mean PDFF of the whole liver segmentation and different axial subvolumes splitting the segmentation from Figure S1 into five equally thick axial volumes. Subvolume 5 has been omitted from this table due to a comparably much lower number of imaging voxels. Generally, the TIMGRECASPR method is slightly overestimating the liver PDFF compared to the Cartesian BH reference scan. The phase correction successfully corrects the overestimation at the upper liver lobe. However, in many subjects it leads to a slight overestimation of the lower liver lobe. This only contributes to a lesser extent to the total mean PDFF due to its smaller volume.

| Whole Liver PDFF |  | BH reference | Self-nav. recon | Self-nav. recon, phase corr |
| --- | --- | --- | --- | --- |
| Volunteer 1 | **Mean PDFF**  Subvolume 1  Subvolume 2  Subvolume 3  Subvolume 4 | **2.1% ± 3.0%** | **3.1% ± 4.8%**  5.0% ± 7.3%  2.2% ± 4.4%  2.3% ± 3.6%  2.2% ± 3.8% | **2.4% ± 4.6%**  1.8% ± 5.0%  2.0% ± 3.4%  2.5% ± 3.4%  3.2% ± 4.0% |
| Volunteer 2 | **Mean PDFF**  Subvolume 1  Subvolume 2  Subvolume 3  Subvolume 4 | **3.0% ± 6.0%** | **4.0% ± 12.9%**  4.9% ± 27.8%  4.0% ± 7.3%  3.8% ± 6.4%  3.7% ± 6.6% | **3.5% ± 7.2%**  3.7% ± 9.2%  3.1% ± 7.1%  3.5% ± 6.5%  4.2% ± 6.6% |
| Volunteer 3 | **Mean PDFF**  Subvolume 1  Subvolume 2  Subvolume 3  Subvolume 4 | **3.0% ± 7.0%** | **3.9% ± 8.4%**  4.6% ± 8.3%  4.4% ± 10.6%  3.4% ± 6.0%  3.0% ± 6.5% | **3.7% ± 8.5%**  3.3% ± 8.7%  3.6% ± 10.8%  3.5% ± 6.0%  4.2% ± 6.3% |
| Volunteer 4 | **Mean PDFF**  Subvolume 1  Subvolume 2  Subvolume 3  Subvolume 4 | **2.0% ± 4.2%** | **3.2% ± 8.4%**  3.7% ± 8.4%  3.7% ± 12.1%  2.9% ± 4.7%  2.7% ± 5.6% | **2.8% ± 6.1%**  1.2% ± 6.7%  2.5% ± 5.6%  2.7% ± 4.0%  3.5% ± 5.0% |
| Volunteer 5 | **Mean PDFF**  Subvolume 1  Subvolume 2  Subvolume 3  Subvolume 4 | **2.1% ± 4.7%** | **3.1% ± 6.1%**  3.7% ± 8.0%  3.3% ± 4.9%  2.7% ± 4.2%  2.2% ± 4.0% | **2.9% ± 6.1%**  2.4% ± 7.7%  2.7% ± 4.9%  2.7% ± 4.2%  2.8% ± 4.0% |

Table S2: Whole liver segmentation *T2** results for five volunteers using the BH reference scan and a self-navigated six echo TIMGRECASPR reconstruction with the proposed phase corrections. The TIMGRECASPR scan was performed three times to check for short-term variability. The mean bias was calculated relative to the BH reference scan. Across all volunteers, the mean absolute *T2** bias was 1.2 ms with wSD = 0.48 ms.

| Whole Liver T_2_* |  | Scan 1 | Scan 2 | Scan 3 | Mean bias [T_2_* ms] |
| --- | --- | --- | --- | --- | --- |
| Volunteer 1 | BH reference | 30.1ms ± 12.4ms |  |  |  |
|  | Self-nav. recon, phase corr | 31.7ms ± 19.5ms | 32.2ms ± 19.5ms | 31.6ms ± 16.9ms | 1.7 |
| Volunteer 2 | BH reference | 21.7ms ± 9.4ms |  |  |  |
|  | Self-nav. recon, phase corr | 20.6ms ± 10.7ms | 21.2ms ± 11.8ms | 20.1ms ± 11.4ms | - 1.1 |
| Volunteer 3 | BH reference | 27.6ms ± 14.8ms |  |  |  |
|  | Self-nav. recon, phase corr | 27.4ms ± 17.9ms | 27.5ms ± 16.0ms | 27.8ms ± 14.4ms | 0.0 |
| Volunteer 4 | BH reference | 31.8ms ± 12.8ms |  |  |  |
|  | Self-nav. recon, phase corr | 31.9ms ± 19.8ms | 32.7ms ± 19.8ms | 32.6ms ± 20.9ms | 0.6 |
| Volunteer 5 | BH reference | 27.2ms ± 15.7ms |  |  |  |
|  | Self-nav. recon, phase corr | 24.2ms ± 13.7ms | 25.4ms ± 15.5ms | 24.1ms ± 14.0ms | - 2.6 |

Table S3: Mean *T_2_** of the whole liver segmentation and different axial subvolumes splitting the segmentation from Figure S1 into five equally thick axial volumes. Subvolume 5 has been omitted from this table due to a comparably much lower number of imaging voxels. Generally, agreement between the TIMGRECASPR and the Cartesian BH reference scan is quite well throughout the whole imaging volume. Standard deviations of the *T_2_** do not differ significantly throughout the subvolumes. This means that the proposed motion self-navigation has been effective in reducing motion artifacts particularly at the upper liver lobe.

| Whole Liver T_2_* |  | BH reference | Self-nav. recon, phase corr |
| --- | --- | --- | --- |
| Volunteer 1 | **Mean T_2_***  Subvolume 1  Subvolume 2  Subvolume 3  Subvolume 4 | **30.1ms ± 12.4ms** | **31.7ms ± 19.5ms** 30.0ms ± 20.1ms  33.4ms ± 20.2ms  30.6ms ± 18.2ms  31.8ms ± 20.1ms |
| Volunteer 2 | **Mean T_2_***  Subvolume 1  Subvolume 2  Subvolume 3  Subvolume 4 | **21.7ms ± 9.4ms** | **20.6ms ± 10.7ms** 19.7ms ± 14.2ms  20.7ms ± 11.7ms  21.2ms ± 8.2ms  20.7ms ± 7.8ms |
| Volunteer 3 | **Mean T_2_***  Subvolume 1  Subvolume 2  Subvolume 3  Subvolume 4 | **27.6ms ± 14.8ms** | **27.4ms ± 17.9ms** 24.6ms ± 21.3ms  28.4ms ± 18.0ms  28.8ms ± 14.8ms  27.1ms ± 18.4ms |
| Volunteer 4 | **Mean T_2_***  Subvolume 1  Subvolume 2  Subvolume 3  Subvolume 4 | **31.8ms ± 12.8ms** | **31.9ms ± 19.8ms** 35.0ms ± 23.0ms  33.3ms ± 20.1ms  31.9ms ± 17.1ms  28.8ms ± 20.7ms |
| Volunteer 5 | **Mean T_2_***  Subvolume 1  Subvolume 2  Subvolume 3  Subvolume 4 | **27.2ms ± 15.7ms** | **24.2ms ± 13.7ms** 23.0ms ± 11.7ms  23.7ms ± 13.5ms  23.7ms ± 12.5ms  26.0ms ± 16.1ms |

Table S4: Whole pancreas segmentation PDFF results for five volunteers using the BH reference scan and a self-navigated six echo TIMGRECASPR reconstruction with the proposed phase corrections. Pancreatic segmentations were obtained from the TotalVibeSegmentator on a previously reconstructed water image (reported DICE coefficients for pancreatic segmentations 0.73-0.81). The TIMGRECASPR scan was performed three times to check for short-term variability. The mean bias was calculated relative to the BH reference scan. Across all volunteers, the mean absolute PDFF bias was 1.1 % with wSD = 0.37 %.

| Whole Pancreas PDFF |  | Scan 1 | Scan 2 | Scan 3 | Mean bias [PDFF %] |
| --- | --- | --- | --- | --- | --- |
| Volunteer 1 | BH reference | 3.4% ± 5.7% |  |  |  |
|  | Self-nav. recon, phase corr | 2.7% ± 3.8% | 2.6% ± 3.6% | 2.8% ± 3.6% | - 0.7 |
| Volunteer 2 | BH reference | 5.8% ± 10.5% |  |  |  |
|  | Self-nav. recon, phase corr | 6.5% ± 6.4% | 6.1% ± 6.0% | 6.9% ± 6.3% | 0.7 |
| Volunteer 3 | BH reference | 4.3% ± 9.1% |  |  |  |
|  | Self-nav. recon, phase corr | 5.7% ± 9.2% | 5.7% ± 8.8% | 5.7% ± 9.5% | 1.4 |
| Volunteer 4 | BH reference | 3.1% ± 5.6% |  |  |  |
|  | Self-nav. recon, phase corr | 4.9% ± 10.6% | 4.1% ± 10.8% | 5.5% ± 11.1% | 1.7 |
| Volunteer 5 | BH reference | 9.7% ± 16.5% |  |  |  |
|  | Self-nav. recon, phase corr | 10.7% ± 16.1% | 10.8% ± 15.6% | 10.5% ± 18.1% | 1.0 |
